# ZBTB40 is a telomere-associated protein and protects telomeres in human ALT cells

**DOI:** 10.1016/j.jbc.2023.105053

**Published:** 2023-07-15

**Authors:** Mingqing Zhou, Yinghong Cui, Shanru Zuo, Qiyao Peng, Yucong Liu, Xueguang Li, Yide Yang, Quanze He, Xing Yu, Junhua Zhou, Zuping He, Quanyuan He

**Affiliations:** 1 The Key Laboratory of Model Animals and Stem Cell Biology in Hunan Province, Hunan Normal University School of Medicine, Changsha, Hunan, China; 2 Center for Reproduction and Genetics, The Affiliated Suzhou Hospital of Nanjing Medical University, Suzhou, Jiangsu, China

**Keywords:** ZBTB40, telomere, ALT, APBs, DNA damage

## Abstract

Alternative lengthening of telomeres (ALTs) mechanism is activated in some somatic, germ cells, and human cancer cells. However, the key regulators and mechanisms of the ALT pathway remain elusive. Here we demonstrated that ZBTB40 is a novel telomere-associated protein and binds to telomeric dsDNA through its N-terminal BTB (BR-C, ttk and bab) or POZ (Pox virus and Zinc finger) domain in ALT cells. Notably, the knockout or knockdown of ZBTB40 resulted in the telomere dysfunction–induced foci and telomere lengthening in the ALT cells. The results also show that ZBTB40 is associated with ALT-associated promyelocytic leukemia nuclear bodies, and the loss of ZBTB40 induces the accumulation of the ALT-associated promyelocytic leukemia nuclear bodies in U2OS cells. Taken together, our results implicate that ZBTB40 is a key player of telomere protection and telomere lengthening regulation in human ALT cells.

Telomeres are DNA and protein complexes located at the termini of chromosomes, and they are essential for the homeostasis of chromosomes and genome integrity (1). Dysfunction of telomeres has been linked to the cell senescence, aging, and many kinds of human diseases (2). Normally, telomeric DNA is encapsulated by a variety of protein complexes, which protects them from being misidentified as DNA double-strand breaks and maintains the proper length of telomeric DNA by telomerase or alternative lengthening of telomere (ALT) pathways (1, 3, 4). Most of cells depend on telomerase reverse transcription to lengthen telomeres (2, 5, 6). Several normal cells and 10% to 15% of cancer cells elongate their telomeres *via* the ALT mechanism that is based on DNA homologous recombination (5, 7, 8) ALT cells have significantly heterogeneous telomeres in length (between 3 kb and 50 kb), and they contain ALT-associated promyelocytic leukemia (PML) body (APB). Recent studies have shown that certain cancer cells neither express telomerase nor activate ALT pathway. However, the molecular mechanisms underlying the telomere length maintenance remain unclear (9, 10).

Zinc finger and BTB (BR-C, ttk and bab) or POZ (Pox virus and Zinc finger) domain (also known as the poxvirus and zinc finger domain,) (ZBTB) genes comprise a large transcription factor family (11, 12), which is featured by a N-terminal BTB/poxvirus and zinc finger domain in protein interactions and several C-terminal C2H2/Kruppel-type Zinc finger responsible for DNA binding. ZBTB genes are important players in various kinds of biological processes, including transcriptional inhibition (13), cytoskeleton regulation (14), tetramerization, gating of ion channels (15), and protein ubiquitination/degradation (16, 17, 18). Many members of this family, for example, BCL6 (19, 20, 21), PLZF (22, 23), cancer hypermethylation 1 (HIC-1) (24, 25, 26), and telomere-associated zinc finger protein TZAP (ZBTB48) (27, 28, 29), are associated with cancer.

Zinc finger and BTB domain-containing 40 (ZBTB40), also known as ZNF923, is the longest member of the ZBTB family, and it is conserved in evolution (30, 31). Recent studies have reported that ZBTB40 promotes the generation of human osteoblasts and reduces the susceptibility to osteoporosis (32, 33). ZBTB40 has been shown to be expressed in the male germ cells and involved in spermatogenesis in *Epinephelus coioides* (34). lncRNA ZBTB40-IT1 has the opposite and antagonistic effect to ZBTB40 protein (35). However, the specific functions of ZBTB40 in human cells remain elucidated.

In this study, we have demonstrated that ZBTB40 is a telomere-associated protein (TAP). The binding of ZBTB40 to telomeric DNA depends primarily on the BTB domain and Zinc fingers. Knockout or knockdown of ZBTB40 in the ALT cells results in telomere dysfunction–induced foci (TIF) and abnormal telomere lengthening. ZBTB40 is also associated with APBs in the U2OS cells, and the knockout of ZBTB40 results in the accumulation of APBs, suggesting its role in telomere recombination regulation. Therefore, our study thus shed novel insight into the mechanisms underlying the telomere length maintenance in ALT cells.

## Results

### ZBTB40 is a TAP in the ALT cells

**Figure 1:**
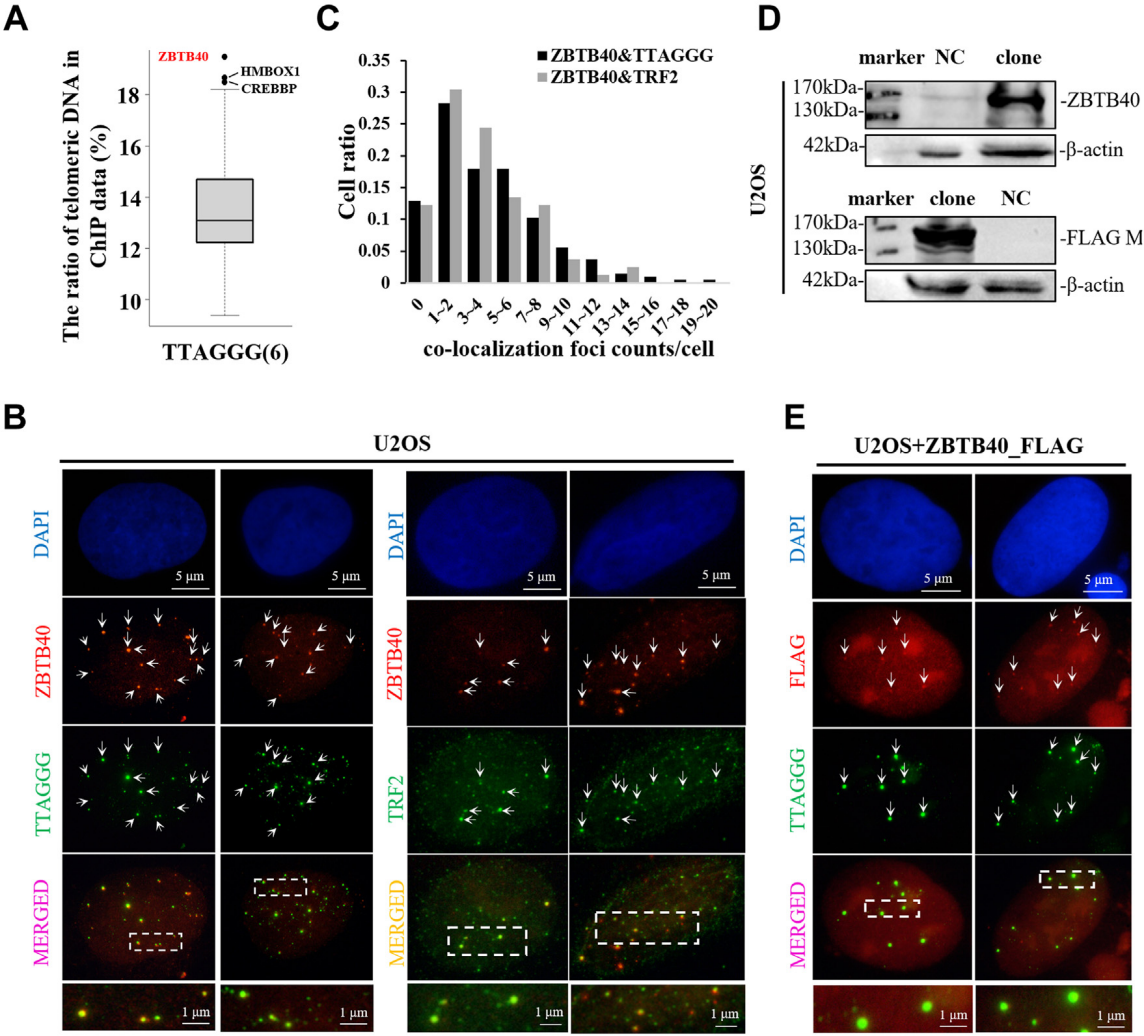
**ZBTB40 is a telomere-associated protein.***A*, the *box plot* showed the ratios of telomeric DNA in chromatin immunoprecipitation (ChIP)-seq data of 127 nuclear proteins in the K562 cells. ZBTB40 and two known TAPs (HMOBOX1 and CREBBP) were labeled. *B*, representative FISH revealed the colocalization of ZBTB40 with TTAGGG/TRF2 in the U2OS cells. The nuclei and target proteins were stained with ZBTB40 (*red fluorescence*), telomeric peptide nucleic acid and TRF2 (*green fluorescence*), and 4′,6-diamidino-2-phenylindole (DAPI) (*blue fluorescence*). The *bottom* of each column was the enlarged field of the view of the area delineated by a *dotted rectangle*. *C*, statistical analysis of Figure 1*B*. Histogram illustrated the numbers of colocalized foci of ZBTB40 and TTAGGG/TRF2 in nuclei of the U2OS cells. *D*, overexpression of ZBTB40 with FLAG tag in the U2OS cells was demonstrated by Western blots. *E*, the colocalization of exogenously expressed ZBTB40 (FLAG in *red fluorescence*) with telomeres in the U2OS cell nuclei. The *bottom* photograph of each column was the enlarged field view of the area delineated by a *dotted rectangle* in related merged image. The *white arrows* in (*B* and *E*) indicated the colocalized foci. ZBTB, Zinc finger and BTB.

To screen novel repetitive element–associated proteins, we downloaded 377 chromatin immunoprecipitation (ChIP)-Seq data of 127 nuclear proteins and eight histone markers in the K562 cells (Fig. 1*A* and Table S9) and calculated the ratios of repetitive reads to the total aligned reads in the ChIP data. For telomeric repeats (TTAGGG_6_), several known TAPs, for example, CREBBP (36) and HMBOX1 (37), were successfully identified, which validated the potential of our screening for novel TAPs identification. Among the TAPs, ZBTB40 was the top one candidate (Fig. 1*A*), which strongly reflects that ZBTB40 is a potential TAP.

To verify the telomeric association of ZBTB40 *in vivo*, we performed fluorescence FISH and immunofluorescence (IF) using telomeric peptide nucleic acid probes (green fluorescence), TRF2 antibody (green fluorescence), and ZBTB40 antibody (red fluorescence) to detect the colocalization of ZBTB40 and telomere in two ALT cells (the U2OS and ZOS cells) (Figs. 1*B* and S1). We revealed that the colocalization foci of ZBTB40 with telomeres were detected in more than 90% of the U2OS cells using both FISH and IF methods (Fig. 1*B*). For the U2OS cells, about 30% of the cell nuclei had 5 to 8 cofoci, and about 5% of the nuclei possessed more than ten cofoci (Fig. 1*C*). To determine whether these foci were derived from ZBTB40, we transiently expressed FLAG-tagged ZBTB40 in the U2OS cells and detected the colocalization signal using FLAG antibody. The expression of FLAG-ZBTB40 was verified by Western blots (Fig. 1*D*) and FISH (Fig. 1*E*). Taken together, these results indicate that ZBTB40 is a TAP in the ALT cells.

Next, we asked whether ZBTB40 could be associated with telomeres in the non-ALT cells. We performed IF–IF experiments in HeLa cells (non-ALT cells) and the U2OS cells (ALT cells) (Fig. S2*A*). Notably, we found that the colocalization foci of ZBTB40 with telomeres were detected in only about 40% of HeLa cells with the significantly less frequency than in the U2OS cells (Chi-squared test *p* < 0.001) (Fig. S2*B*). These results suggest that ZBTB40 plays an essential role in telomere regulation in the ALT cells.

### BTB domain mediates the binding of ZBTB40 to telomeric DNA

**Figure 2:**
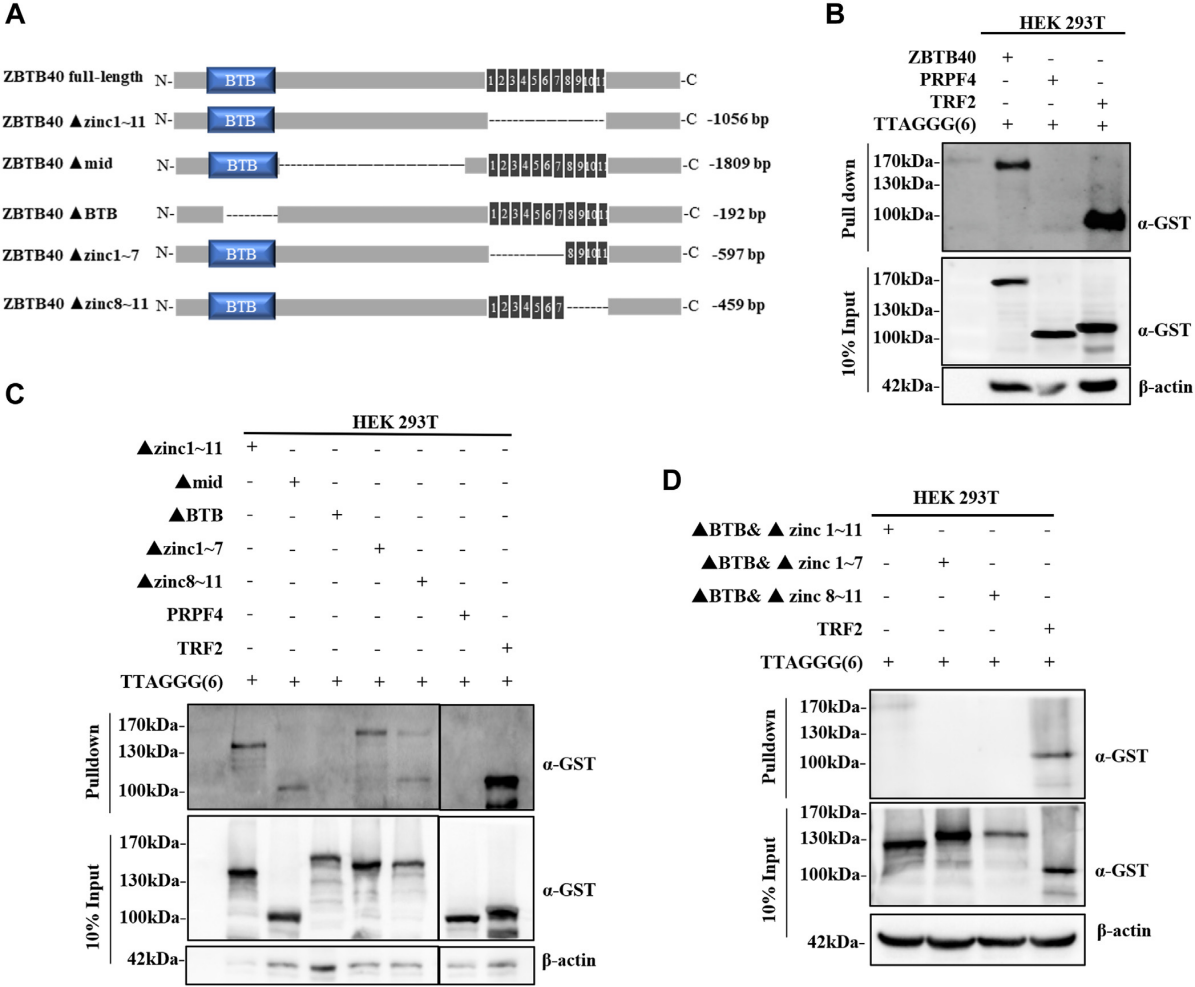
**BTB domain and Zinc finger domain (8–11) are responsible for the telomere DNA binding.***A*, the schematic diagram indicated the design of ZBTB40 fragment deletion mutants. The lengths of deletion region in amino acid were shown at the *right side*. *B*, the DNA pull-down assay to detect the binding of ZBTB40 to telomeric DNA. The cell lysates of HEK 293T cells stably expressed ZBTB40-GST, TRF2-GST (positive control), and PRPF4-GST (negative control) were incubated with streptavidin beads which were bound by biotin-labeled telomeric DNA. The beads-bound proteins were detected by Western blots. *C* and *D*, the binding affinity of ZBTB40-GST mutants to telomeric DNA detected by the DNA pull-down assays. GST, glutathione-*S*-transferase; ZBTB, Zinc finger and BTB.

Since some ZBTB proteins have the ability to bind to telomeric DNA through BTB and zinc finger domains (27, 29, 38), we hypothesized that ZBTB40 interacts with telomeric DNA with the similar mechanism (Fig. S3). To test this hypothesis, we utilized the streptavidin agarose pull-down assay to detect the binding affinity between double-strand telomeric DNA (TTAGGG_6_) and glutathione-*S*-transferase (GST)-tagged ZBTB40 full-length and mutant proteins (Fig. 2*A*). We examined the importance of several target domains/regions (1 ∼ 11, 1 ∼ 7, and 8 ∼ 11 Zinc finger domains, long mid domain, and BTB domain) for ZBTB40 to bind to telomeres. We found that the full-length GST-ZBTB40 protein could be pulled down by telomeric DNA (Fig. 2*B*) and that the loss of BTB domain disrupted the binding completely (Fig. 2, *C* and *D*). Interestingly, the removal of some or all of zinc finger domains did not affect the binding of GST-ZBTB40 to telomeric DNA (Fig. 2, *C* and *D*), implicating that these domains are not essential for GST-ZBTB40-telomere association. We also investigated the potential association of GST-ZBTB40 with other telomeric proteins. Our co-immunoprecipitation results indicate potential direct or indirect interaction between ZBTB40 and TRF2 (Fig. S4).

### Knockdown or knockout of ZBTB40 leads to telomere dysfunction and apoptosis

**Figure 3:**
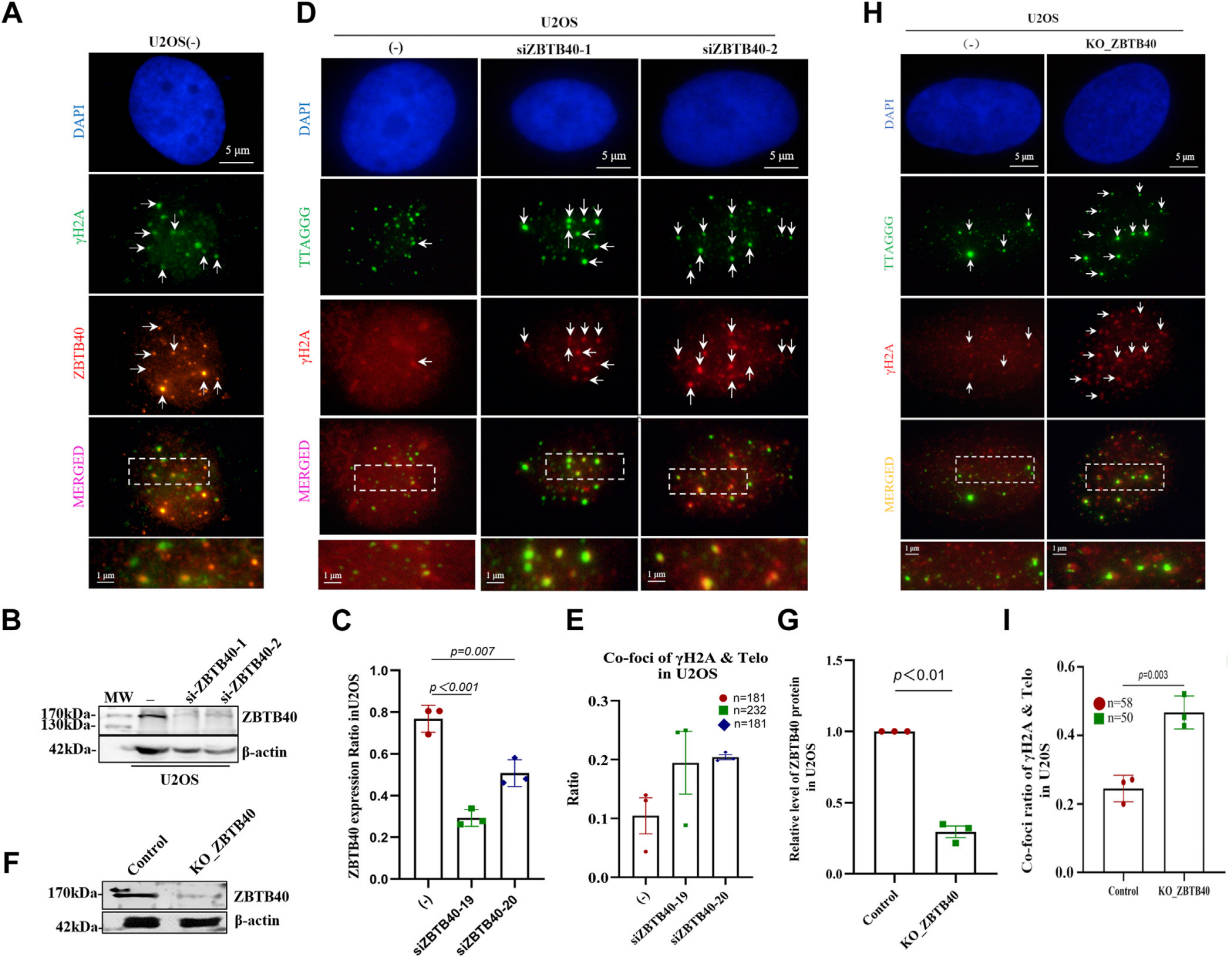
**Loss of ZBTB40 leads to telomere dysfunction and TIF of the U2OS cells.***A*, IF showed the colocalization of ZBTB40 (*red fluorescence*) and γH2A (*green fluorescence*) in the U2OS cells. *B*, Western blots displayed that ZBTB40 protein was reduced by two ZBTB40 siRNAs in the U2OS cells. *C*, ImageJ (https://imagej.nih.gov/ij/) calculated the grayscale value of the immune imprinting strips in Figure (*B*) (three biological repeats). *D*, representative IF-FISH showed the TIFs induced by ZBTB40 knockdown. Telomeric DNA and γH2A were staining as *green fluorescence* and *red fluorescence*, respectively, in the U2OS cells. *White arrows* indicated the colocalization of γH2A and telomere DNA (TIF) in the U2OS cells. *E*, the statistical analysis of the ratio of TIF to telomeres in three independent experiments in Figure (*D*). The numbers of nuclei counted were shown at *right-top corner*, and ∗ indicates *p* < 0.05 in *t* test for three biological experimental repeats. *F*, Western blots revealed the ZBTB40 protein level in ZBTB40 KO U2OS cells. *G*, quantification of Figure (*F*) (three biological repeats). *H*, representative FISH illustrated the TIFs induced by the ZBTB40 KO U2OS cells. *I*, statistic assay of the ratio of γH2A to telomeres in the control and the ZBTB40 KO U2OS cells. For *p* values of *t* tests in all figures: ∗ indicates *p* < 0.05, ∗∗ indicates *p* < 0.01, and ∗∗∗ indicates *p* < 0.001. IF, immunofluorescence; TIF, telomere dysfunction–induced foci; ZBTB, Zinc finger and BTB.

Because ZBTB proteins have been reported to protect telomeres from abnormal DNA damage, we examined whether ZBTB40 is involved in this function. We uncovered the colocalization of ZBTB40 with γ-H2AX in the U2OS and ZOS cells (Figs. 3*A* and S5), suggesting that ZBTB40 is recruited to some DNA damage sites in the ALT cells. We then asked whether the loss of ZBTB40 induces the formation of TIFs (39, 40). The U2OS cells were treated with two ZBTB40 siRNAs for 24 h, which decreased the ZBTB40 level by 20% and 50%, respectively (Fig. 3, *B* and *C*). In the ZBTB40-knockdown U2OS cells, the percentage the TIF-positive cell was 2.5 times higher than vehicle control (*p* < 0.05). To further validate the results, we generated the ZBTB40 KO cells (mixed clone) by CRISPR/CAS9 method (Figs. 3, *F* and *G* and S6). Consistent with our previous results, the TIF assay showed that the ratio of telomeres with TIF in the KO U2OS cell was twice more than negative control (Fig. 3, *H* and *I*). In the ZOS cell, the ratio of TIF was 4-folds higher in ZBTB40 KO ZOS cells than control (Fig. S7). Considered together, these data indicate that the loss of ZBTB40 results in telomere dysfunction and that ZBTB40 is involved in telomere protection in the ALT cells. To investigate whether this telomere dysfunction associated with any deleterious cell phenotype, we checked the apoptosis ratio of WT and ZBTB40-deficient U2OS cells and found that ZBTB40-deficient cells have significantly higher apoptosis ratio than WT cells (Fig. S8).

### Knockdown or knockout of ZBTB40 causes telomere lengthening

**Figure 4:**
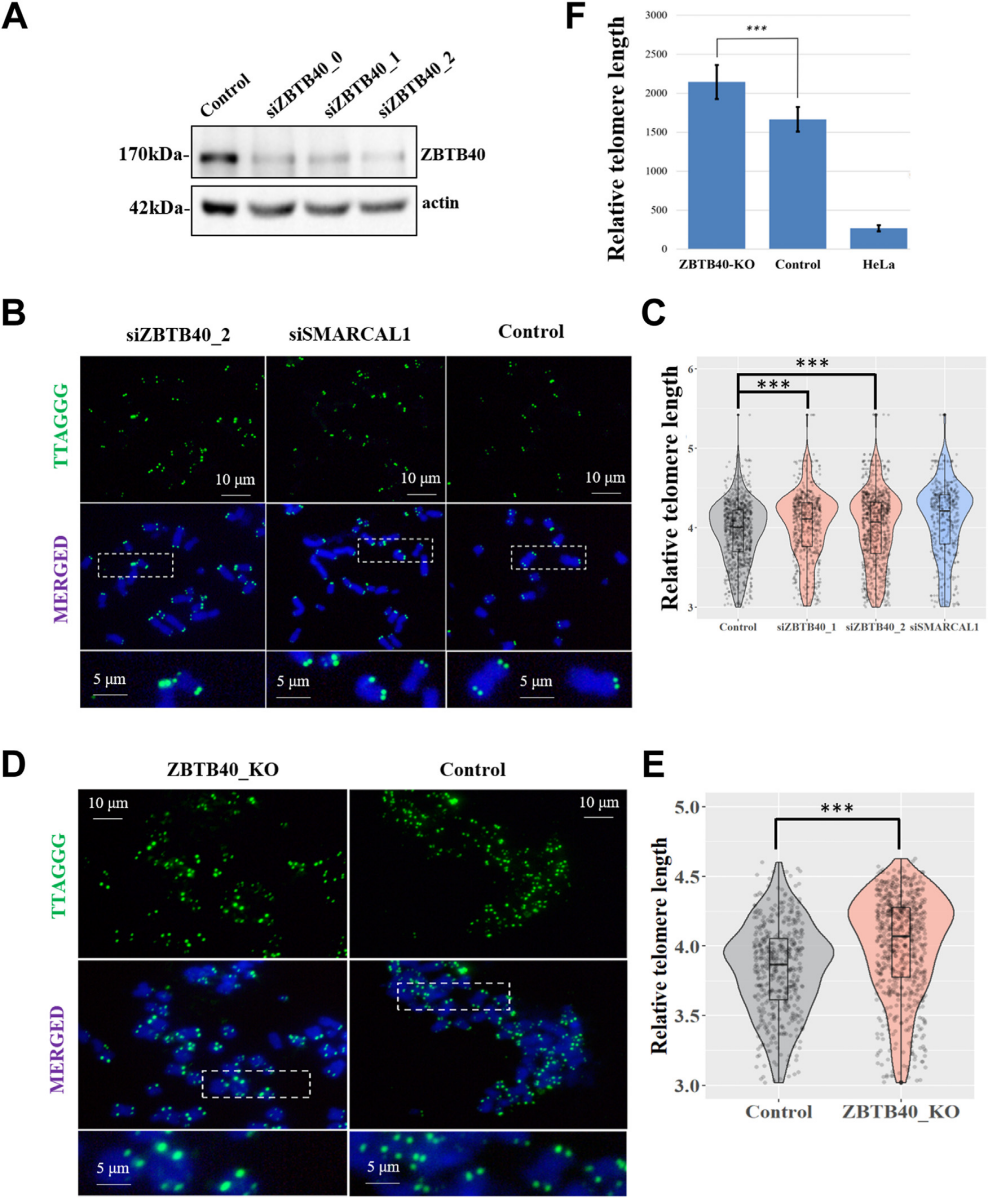
**ZBTB40 regulates telomere length in the U2OS cell.***A*, Western blots showed the knockdown efficiency of three ZBTB40 siRNAs (*top panel*). *B*, representative Q-FISH pictures illustrated telomere foci in M-phase of the U2OS cells with knockdown of target genes (SMARCAL1 as a positive control) and the negative control. *C*, a *violin plot* to present the distribution of relative telomere length in samples of (*B*). *D*, Q-FISH results of the ZBTB40 KO U2OS cells and the negative control. *D*, representative Q-FISH images show telomere foci in M-phase of the U2OS KO cells and WT control. *E*, the *violin plot* showed the distribution of relative telomere length of the samples in the Figure (*D*). *F*, the relative telomere length (T/S ratio) in the KO and control cells measured by qPCR. Wilcoxon one-sided tests were performed to check the statistical significance of the difference and calculate *p* values in figures: ∗ indicates *p* < 0.05, ∗∗ indicates *p* < 0.01, and ∗∗∗ indicates *p* < 0.001. ZBTB, Zinc finger and BTB.

We next checked if ZBTB40 is involved in the regulation of telomere length. We performed the Q-FISH to measure the length of telomeres in the mitotic phase ZBTB40 knockdown and KO U2OS cells (Fig. 4*A*). The value of telomere fluorescence at M phase was measured and transformed to relative length of telomeres by software (Fig. 4*C*). Comparing with the negative control group, the deficiency of ZBTB40 results in longer telomeres (*p* < 0.01) in both ZBTB40 knockdown (Figs. 4*B* and S10*A*) and KO U2OS cells (Figs. 4*D* and S10*B*). The results were further demonstrated by the telomere length quantitative polymerase chain reaction (qPCR) assays (Fig. 4*F*). These data suggest that ZBTB40, like TZAP, is involved in telomere trimming.

### Loss of ZBTB40 induces APBs accumulation in the U2OS cells

**Figure 5:**
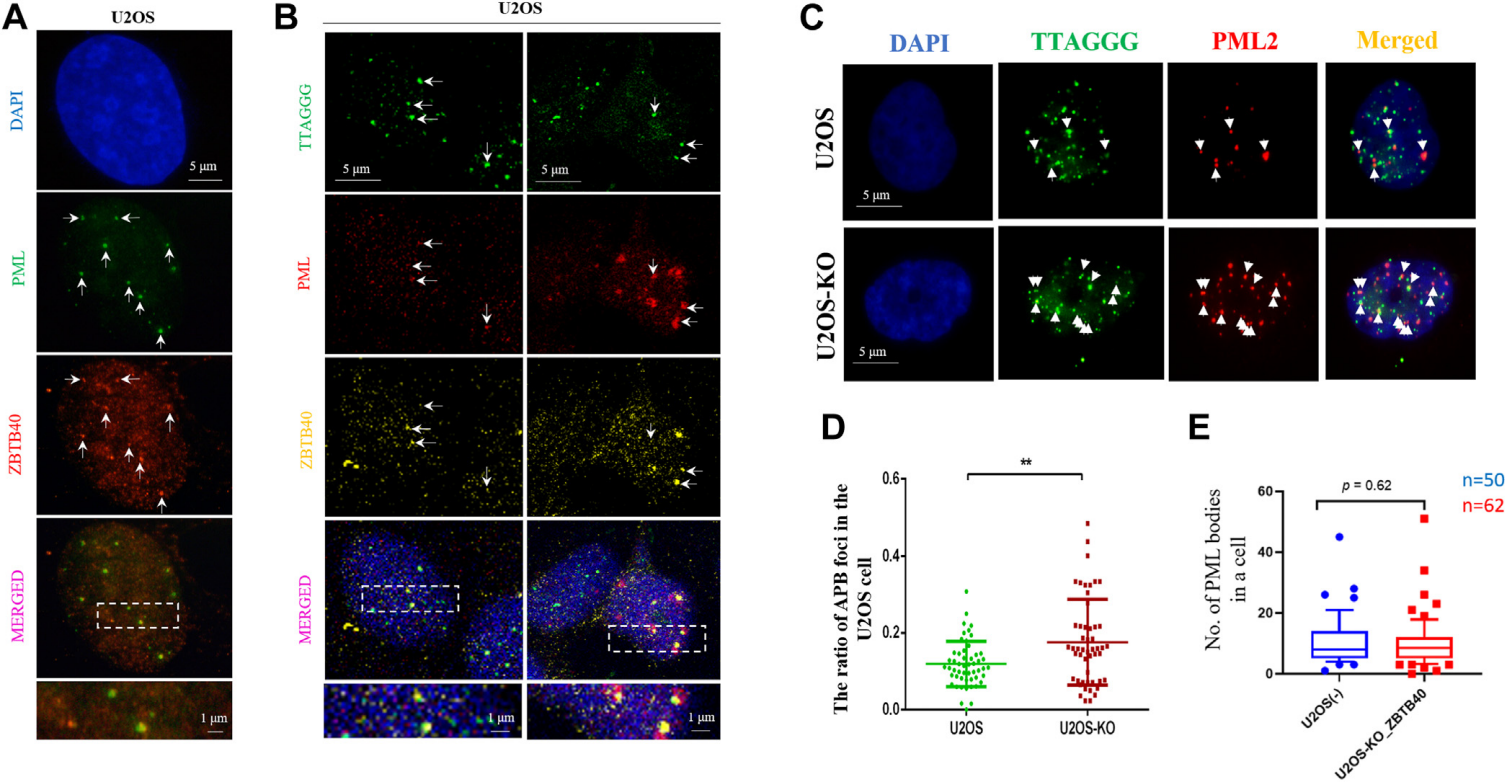
**ZBTB40 is associated with APBs in the U2OS cells.***A*, immunofluorescence illustrated the colocalization of ZBTB40 (*red fluorescence*) and PML (*green fluorescence*) in the U2OS cells. *B*, representative IF-FISH images revealed the colocalization of ZBTB40 (*yellow fluorescence*) with APBs (PML + TTAGGG(n)) in the U2OS cells. The *white arrows* indicated the APBs. *C*, FISH indicated the number of APBs was increased in ZBTB40 KO U2OS cells. *D*, the *box plot* shows the statistical analysis of the ratio of ABPs to telomeres in the Figure (*C*). *E*, the number of PML bodies in WT and the ZBTB40 KO U2OS cells. For *p* values of *t* tests in the figures: ∗∗ denoted *p* < 0.01. APB, ALT-associated promyelocytic leukemia body; IF, immunofluorescence; PML, promyelocytic leukemia; ZBTB, Zinc finger and BTB.

Another hallmark of ALT cells is an increased frequency of PML colocalization with telomeric DNA. These APBs contain telomere clusters and provide a “recombinogenic microenvironment” to promote ALT (41). Using IF method, we found that ZBTB40 was colocalized with PML body in the U2OS cell (Fig. 5*A*). Our IF-FISH further revealed that ZBTB40 could colocalize with APBs in the U2OS cell (Fig. 5*B*). Interestedly, we found that the number of ABPs in ZBTB40 KO U2OS cells was significantly higher than that in the WT cells (Fig. 5, *C* and *D*). However, the loss of ZBTB40 in KO cells did not affect the number of PML bodies (Fig. 5*E*), which ruled out the possibility that the increase of ABPs in the KO cells resulted from the PML bodies increase. As the APB bodies are the hubs of telomere recombination (41), our results suggested that ZBTB40 may negatively regulate the formation of the APBs and therefore repress the recombination of telomeres. As APBs are formed in cell cycle–dependent manner during S phase in ALT cells (42), we compared the S phase distribution of ZBTB40 KO and WT U2OS cells and found that no significant difference between ZBTB40 KO and WT U2OS cells in term of S phase (Fig. S9), suggesting that APBs accumulation is not simply caused by the possible cell cycle change in ZBTB40 KO cells.

## Discussions

ZBTB40 gene encodes the longest protein in the ZBTB family, and it is evolutionarily conserved from Protostomia to human (30, 31). As a transcriptional factor, the molecular and physiologic function of ZBTB40 remains unclear (34). Recently, two members of ZBTB family (TZAP and ZBTB10) have been found to be involved in telomere trimming and regulation, which sheds new light on ZBTB genes’ functions (28, 38). Especially, TZAP bindings to telomeres with its zinc finger domain, recruits TRF1 and TRF2 and prevents enzyme from shorting telomere and therefore promotes telomere lengthening (28). ZBTB10 has been shown to repress expression of telomerase and interact with TRF1 to regulate telomere length (38). Here, our study added a new member to the gene list and showed that ZBTB40 is essential for telomere protection and lengthening regulation in ALT cells. We found that loss of ZBTB40 results in APBs accumulation in the ALT cells. As APBs have long been considered as telomere recombination centers (41), our results suggest the possibility that ZBTB40 prohibits the telomere lengthening in ALT cells by repressing APB formation.

Although major telomeric function of ZBTB40 was focused on the ALT cells in this study, we did not rule out the possibility that ZBTB40 may also regulate telomeres in the non-ALT cells, as there is evidence supporting this potential. For example, the ChIP-Seq data indicate the association between ZBTB40 and telomeres in the K562 cells, and the ZBTB40/telomere colocalization foci were observed in the HeLa cells in this study. Both the K562 and HeLa cells are telomerase-positive cells. The potential roles of ZBTB40 in telomere regulation in the non-ALT cells remain to be further clarified.

The BTB domain usually mediates protein interaction instead of DNA binding. However, our DNA pull-down assay reveals that the BTB domain rather than the Zin fingers is required for ZBTB40 binding to telomeric DNA. Interestingly, the BTB domain of TZAP has been reported to be essential for telomeric recruitment, and the BTB domains from ZBTB family can form the homodimers (12). It is possible that ZBTB40 is recruited to telomeres through the interaction with other telomere-associated ZBTB proteins (*e.g.*, TAZP). Nevertheless, no ZBTB protein or other TAP has been found in the interaction partner of ZBTB40 in protein–protein interaction database (*e.g.*, STRING (43) and BioGRID (44)). Interestedly, the association of ZBTB40 with TRF2 identified by our study suggests another potential mechanism by telomeric recruitment of ZBTB40. Whether BTB domain can directly bind to telomeric DNA or interacts with other telomere-binding proteins needs to be further explored.

On the other side, similar with TZAP, ZBTB40 prefers to bind to the long telomeres (Fig. S11), and it is essential for repressing telomere lengthening in the ALT cell to prohibit telomeres from getting extreme long. The underlying mechanisms of these ZBTB proteins contributing to the telomere lengthening inhibition await further examination. Two candidate models are suggested as follows: (1) DNA damage on long telomeres triggers the telomeric recruitment of ZBTB proteins, which associate with the enzyme to cause telomere trimming and (2) ZBTB proteins repress telomere lengthening by inhibiting APB formation and telomeric recombination in the ALT cells, which was supported by our data.

Recently, we also found that *Zbtb40* protects telomeres from DNA damage and modulates telomere length in mouse spermatocytes and apoptosis of male germ cells (45). The testis and cauda epididymis body weights of *Zbtb40*^*+/−*^ mice were significantly decreased, and the sperm of *Zbtb40*^*+/−*^ mice were assumed as abnormal flagellum acrosome, which leads to male infertility. These results are consistent with our founding in ALT cells and highlighted the physiological function of ZBTB40 in mammals.

## Experimental procedures

### Cell culture and transfection

Cell lines were cultured in Dulbecco's modified Eagle's medium (c11995500BT, Thermo Fisher Scientific) supplemented with 10% fetal bovine serum (10270106, Thermo Fisher Scientific) and 1% penicillin-streptomycin (15140122, Thermo Fisher Scientific). Plasmids or siRNAs were transfected into the cells using the Lipofectamine 2000 (11668019, Thermo Fisher Scientific) according to manufacturer’s instructions, and the medium was changed at 6 to 8 h after transfection. The cells were harvested at 48 to 72 h after transfection for the further studies. The sequences of Stealth RNAi siRNAs for ZBTB40 (10620318, 10620319, 10620320, Thermo Fisher Scientific) were shown in Table S1.

### ChIP-Seq data analysis

In total, 377 ChIP-Seq data of 127 nuclear proteins and eight histone markers in K562 cells were downloaded from ENCODE database (https://www.encodeproject.org/) (Table S9). All FASTQ data should have at least 20 M reads with read lengths over 37 bp. These FASTQ files were mapped to Hg38 human genome using BWA (46) and SAMtools (47). Then, a homemade python script was applied to search reads containing repetitive sequences with no mismatches. For telomere, the repetitive sequence is (TTAGGG)_6_. The binding intensities of target genes were measured by the ratios of repeats containing reads to total mapped reads in the FASTQ files.

### Western blots

Cells were lysed with ice cold NETN (100 mM NaCl, 20 mM Tris–HCl pH8.0, 5 mM EDTA, 0.5% NP-40 with protease inhibitors and DTT). The cell lysates were denatured for 10 min at 100 °C and resolved by 8% to 16% SDS-PAGE gels and transferred to HYBOND-N+ membranes (RPN203B, GE life) at 200 mA for 2 h. The membranes were blocked by 5% (w/v) nonfat milk (DH220-3, DING GUO) for 1 h at room temperature (RT), and they were incubated with primary antibodies diluted in QuickBlock Western antibody dilution solution (P0256, Beyotime) for 2 h at RT or overnight at 4 °C. The detailed information on the antibodies was included in Tables S5 and S6. After three washes in Tris-buffered saline with 0.1% Tween® 20 detergent (150 mM NaCl, 20 mM Tris–HCl, pH8.0, 0.1% Tween), the membranes were incubated with secondary antibody for 1 h at RT. Proteins were detected by chemiluminescence using Enhanced Chemiluminescence kit (GE2301, GENVIEW).

### Fluorescence *in situ* hybridization

FISH was conducted pursuant to the method as described previously (48). Briefly, the cells grown on coverslips were treated with 4% paraformaldehyde and permeabilization solution (5% Triton X-100, 20 mM Hepes pH 7.5, 50 mM NaCl, 3 mM MgCl_2_, 300 mM Sucrose), and they were incubated with blocking solution (0.1% bovine serum albumin, 3% goat serum/fetal bovine serum in PBS) for 30 min and then with primary antibodies (Table S5) in blocking solution for 1 h at RT. Coverslips were washed with PBS three times and incubated with secondary antibodies in blocking solution for 30 min. Cells were then washed with PBS three times.

FISH was performed in terms of the following procedure. After the last wash by PBS, coverslips were dehydrated consecutively in 70%, 95%, and 100% ethanol for 5 min each. FITC-OO-[CCCTAA]3–labeled peptide nucleic acid probe (PANAGENE) hybridizing solution (70% formamide, 10% blocking reagent (Roche), 1 M Tris–HCl pH7.4, buffer MgCl_2_ (25 mM MgCl_2_, 9 mM citric acid, 82 mM Na2HPO4) was added to the coverslips for denaturing at 80 °C for 5 min, and they were hybridized for 2 h at RT in the dark. The coverslips were washed with wash I solution (70% formamide, 10 mM Tris–HCl, pH 7–7.5) and wash II solution (0.15 M NaCl, 100 mM Tris–HCl, pH 7–7.5, 0.08% Tween) for 15 min each, and they were dehydrated consecutively in 70%, 95%, and 100% ethanol for 5 min each. The cells were counterstained with 4′,6-diamidino-2-phenylindole (H1800, VECTASHIED), and digital images were captured on Zeiss M1.

### Quantitative FISH

The Q-FISH was conducted in terms of the protocol as described previously (48). Cells were cultured with the 6-well plates for 24 h before nocodazole treatment, and 0.5 μg/ml nocodazole was added to cell medium to arrest mitosis for at least 1.5 h. Hypotonic solution in 10 ml 0.075 M KCl and the fixative medium (methanol/glacial acetic acid 3:1, fresh and cool down at −20 °C) were added to the cells to fix them for 30 min. The supernatant was removed, and the cells were resuspended with 0.25 to 0.5 ml fixative medium and placed to slides by cytospin. The cells were dehydrated by 70%, 95%, and 100% ethanol, and the slides were treated with RNase (R6148, Sigma, 1:100 dilution) PBS solution and pepsin solution (50 ml 10 mM HCl, 125 μl 1% pepsin) for 10 min. The slides were washed with 2× saline-sodium citrate buffer, and they were dehydrated by 70%, 95%, and 100% ethanol. FISH hybridization of metaphase spreads (≥30 metaphases per sample) was performed as previously described (49), which was visualized on a Nikon TE200 fluorescence microscope and analyzed with TFL-TELO (Leica Imaging Systems; https://pubmed.ncbi.nlm.nih.gov/10404142/). For chromosomal aberrations and karyotyping, more than 50 metaphases per sample were analyzed (50).

### Telomeric DNA pull-down assay

Telomeric DNA pull-down assay was done according to the method (51) with minor modification. Briefly, 25 μg of the forward and reverse sequence oligonucleotides (Table S2) were diluted in annealing buffer (20 mM Tris–HCl, pH7.5, 10 mM MgCl_2_, 100 mM KCl), denatured at 95 °C, and annealed by cooling. Biotin-labeled telomeric DNA and control DNA were immobilized on 500 μg paramagnetic streptavidin beads (Dynabeads MyOne C1, Thermo Fisher Scientific) by rotating for 30 min at RT. Cells were lysed (for a 6-well plate) using 150 μl ice cold NETN (100 mM NaCl, 20 mM Tris–HCl pH8.0, 5 mM EDTA, 0.5% NP-40 with protease inhibitors and DTT). Cell lysates were collected and rotated for 30 min at 4 °C and centrifuged for 3 to 5 min at 10,000 to 14,000*g*, and the supernatant was kept. Subsequently, bait beads were incubated with cell lysates (400–800 μg proteins) by rotating for 2 h at 4 °C. Twenty micrograms of the sheared salmon sperm DNA (Ambion) were added as a competitor for DNA binding. After washes with PBS buffer three times, bound proteins were eluted in 2× SDS loading buffer, boiled for 10 min at 100 °C, and separated by SDS-PAGE. The ZBTB40 mutant plasmids (Fig. 4*A*) with GST tag were expressed in HEK 293T cells, and PRPF4 (an RNA-binding protein, splicing factor (52)) and TRF2 (a component of the sheltering complex) served as the negative control and positive control, respectively.

### ZBTB40 mutation constructs

The ZBTB40 mutation constructs were completed, and the primers used for the mutation construction were shown in Table S3.

### CRISPR/CAS9 targeting strategy for generating ZBTB40 KO cells

ZBTB40 KO cells were generated using CRISPR/Cas9 gene targeting of the U2OS cells. Single guide RNAs (sgRNAs) were cloned into CAG-Cas9-T2A-EGFP-ires-puro (Addgene), and they were electro-transfected to the U2OS cells. Targeting of ZBTB40 in the U2OS cells was carried out by the following sgRNA1: AAGGTTAGAGCATGGAGTCGTGG (exon5) and sgRNA2: ATTAACGTGTGGCTTTCCCAGGG (exon7) (Table S7). The mixed KO cells were amplified and selected by 2 to 10 μg/μl puromycin at 7 days, and single KO clones were picked up and validated using PCR and Western blots. The validation of ZBTB40 knockout in U2OS was performed by target genomic PCR (Table S8).

### Telomere length measurement by qPCR

Monochrome multiplex qPCR was performed as previously described (53) to compare the relative length of telomeres of ZBTB40-KO and the control U2OS cells. Genomic DNA was extracted directly from cells using the QIAamp DNA Mini Kit (Cat. NO. 51340). The cycle threshold (Ct) values were measured for telomeres and albumin (internal control). The relative telomere length was calculated by telomere/single-copy gene (T/S) values with the formula 2^−ΔCt^, where ΔCt = Ct telomere – Ct albumin. The related primers and PCR master mix could be found in Tables S1, S2 and S4.

### Cell apoptosis detection

Cells were stained with adenomatous polyposis (APC)/annexin V and propidium iodide (PI), and the apoptosis rate was detected with flow cytometry. Briefly, U2OS cells and ZBTB40-knockdown U2OS cells were collected and washed twice with PBS. Then cells were resuspended with 100 μl annexin V binding buffer containing 5 μl APC/annexin V and 10 μl PI and were incubated for 15 min at RT in the dark. Four hundred microliters annexin V binding buffer was used to stop staining. And the rate of APC/annexin V-positive of PI-positive cells were detected by flow cytometry (BD Bioscience, FACS CantoII 488N).

### Cell cycle analysis

PI staining was used to detect the cell cycle distribution. According to the manufacturer's instructions, U2OS cells or ZBTB40-knockdown U2OS cells were fixed with 75% cold ethanol overnight at −20 °C and were washed with PBS. Cells were resuspended with 50 μg/ml RNase and incubated for 30 min at 37 °C in the dark. Then, cells were treated with 65 μg/ml PI for 30 min at 4 °C in the dark. The flow cytometry (BD Bioscience, FACS CantoII 488N) was utilized to detect the number of cells at G1, S, and G2/M phases.

### Statistical analysis

The *t* test or single-factor ANOVA analysis was performed using the SPSS statistical software and GraphPad Prism (https://www.graphpad.com/), and *p* < 0.05 indicated that the difference was statistically significant.

## Data availability

All data are contained within the article.

## Supporting information

This article contains supporting information.

## Accession numbers

The accession number list of the ChIP-Seq data used in this paper could be found in Supplemental materials (Table S9).

## Conflict of interest

The authors declare that they have no conflicts of interest with the contents of this article.
