# Supplementary material for: ZBTB40 is a telomere-associated protein and protects telomeres in human ALT cells
Source: J Biol Chem. 2023 Jul 15;299(9):105053. doi: 10.1016/j.jbc.2023.105053 (PMC10480536; doi:10.1016/j.jbc.2023.105053)
Supplement: Supporting Figures S1–S11 and Tables S1–S9 [file mmc1.docx]

**Supplemental Data**

**ZBTB40 is a telomere-associated protein and protects telomeres in human ALT cells**

Mingqing Zhou^1#^, Yinghong Cui^1#^, Shanru Zuo^1^, Qiyao Peng^1^, Yucong Liu^1^, Xueguang Li^1^, Yide Yang^1^, Quanze He^2^, Xing Yu^1^, Junhua Zhou^1^, Zuping He^1*^, Quanyuan He^1*^

**Supplemental Figures 1~11**

Figure S1. ZBTB40 is colocalized with telomers in the ZOS cell.

Figure S2. ZBTB40 prefers to binding to telomeres of the ALT cells.

Figure S3. Comparison of ZBTB40 against ZBTB48 and ZBTB10 in protein domain and sequences.

Figure S4. The CO-IP assays detect the association between ZBTB40 and TRF2.

Figure S5. ZBTB40 is co-localized with γ-H2AX in the ZOS cell.

Figure S6. The generation of the ZBTB40 knockout U2OS cells.

Figure S7. Loss of ZBTB40 leads to telomere dysfunction in the ZOS cells.

Figure S8. The cell apoptois ratio of the WT and ZBTB40 defected U2OS cells.

Figure S9. The cell cycle progress in WT and ZBTB40 defected U2OS cells.

Figure S10. The distribution of relative telomere length in the ZBTB40 defected U2OS cells.

Figure S11. The distribution of telomere length of telomere foci colocalized with or without ZBTB40 in U2OS cells.

**Supplemental Tables 1~9**

Table S1. Sequences of siRNAs

Table S2. Sequences of telomeric oligonucleotides

Table S3. Primers of ZBTB40 mutation constructs

Table S4. Primers of real-time quantitative PCR

Table S5. Primary antibodies utilized in this study

Table S6. Secondary antibodies used in this study

Table S7. gRNAs used in ZBTB40 knockout U2OS cell generation.

Table S8. Primers for ZBTB40 knockout U2OS cell validation

Table S9. Accession IDs of ChIP-Seq data employed in this study

**Supplemental Figures 1-12**

**
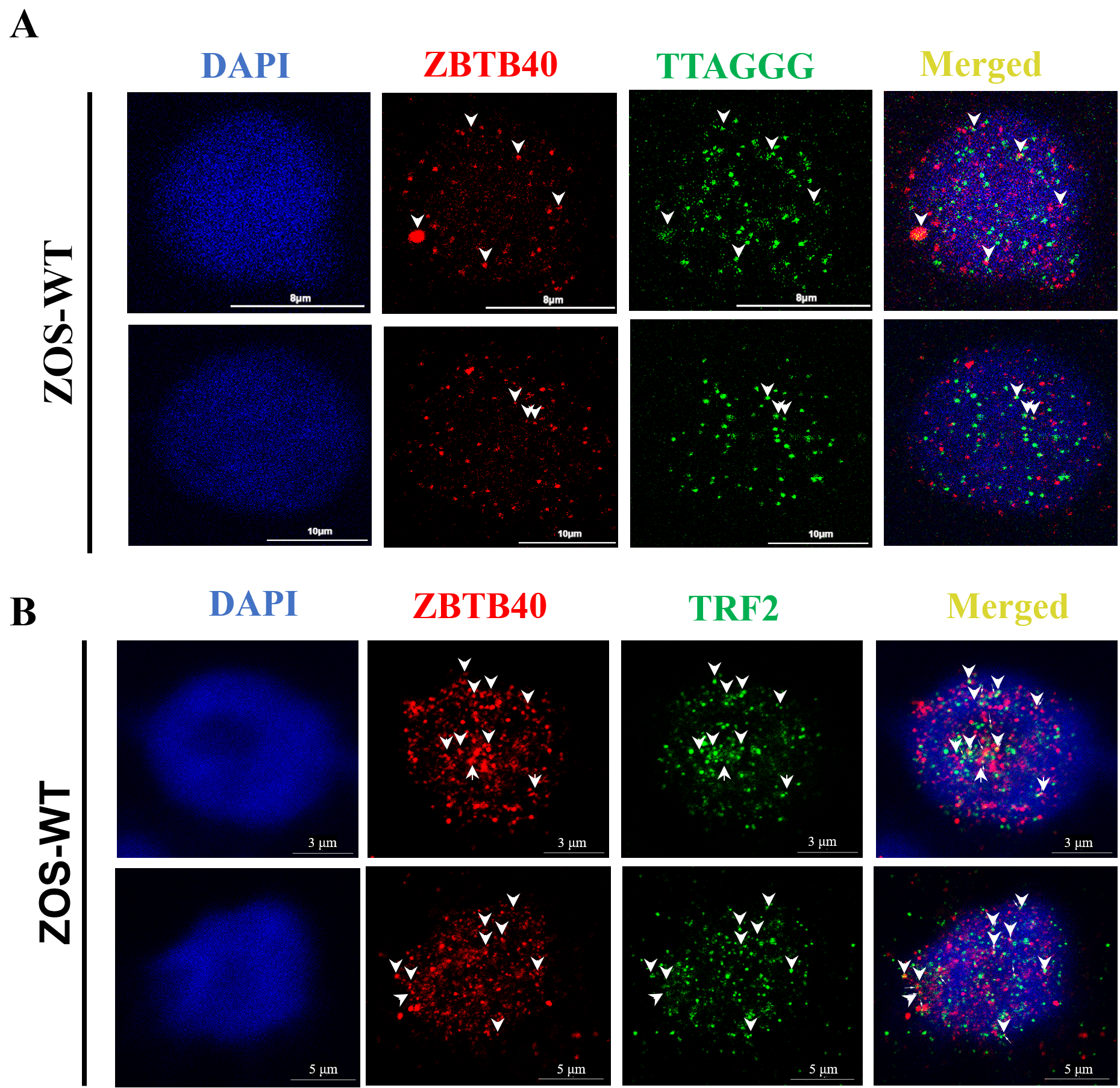
**

**Figure S1. ZBTB40 is co-localized with telomers in the ZOS cells.** A. Representative FISH results showing the co-localization of ZBTB40 (red fluorescence) with telomere (green fluorescence); B. Representative immunofluorescence results displaying the co-localization of ZBTB40 with TRF2 in the ZOS cells. The nuclei and target proteins were stained with DAPI (blue fluorescence), ZBTB40 (red fluorescence), and TRF2 (green) fluorescence. The co-localized foci were indicated by white arrows.

**
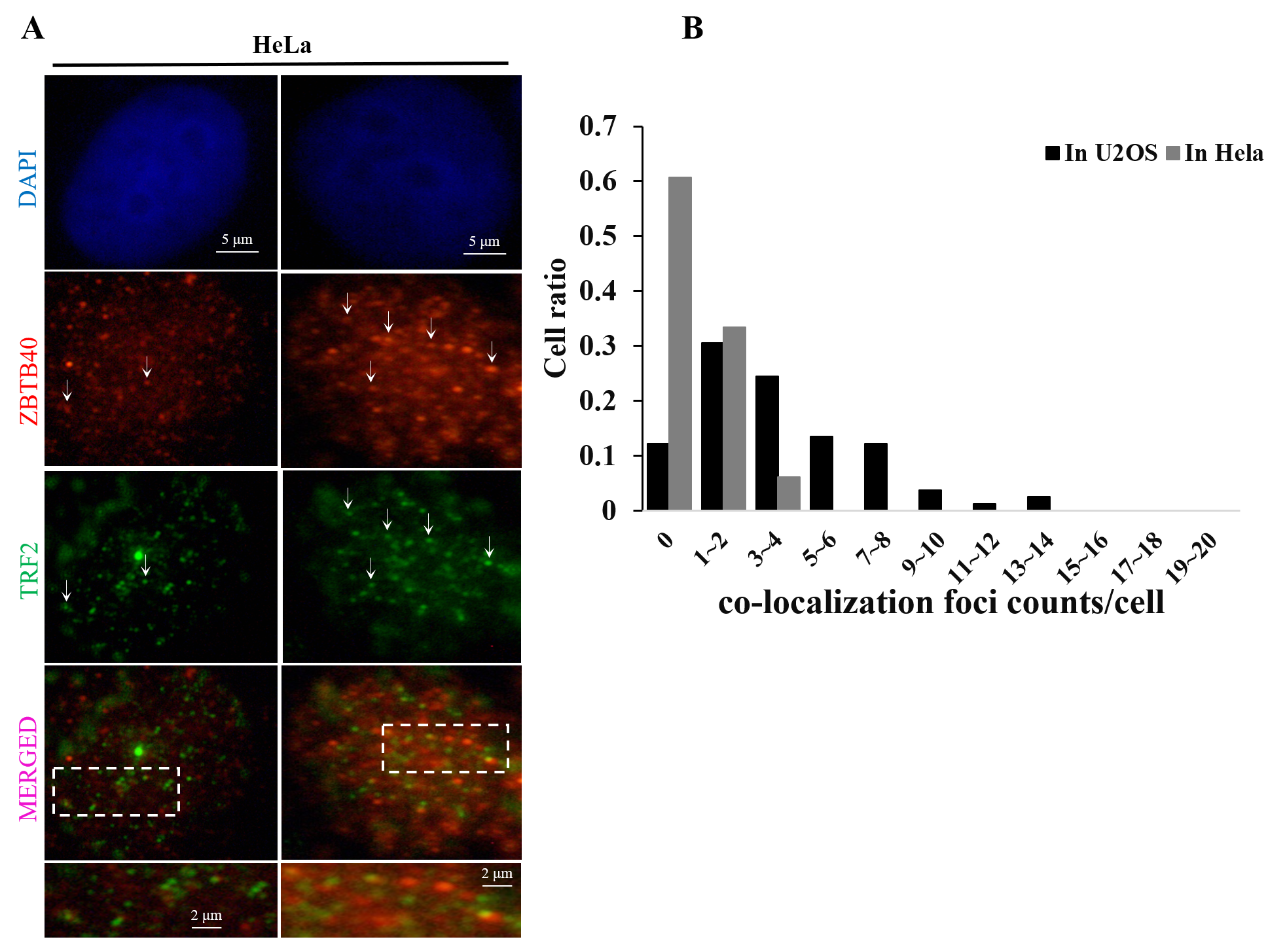
Figure S2. ZBTB40 prefers to binding to telomeres of the ALT cells.** A. Representative immunofluorescence (IF) staining pictures of ZBTB40 and TRF2 in HeLa cells. The nucleus and targeted proteins were stained by DAPI (blue fluorescence), ZBTB40 antibody (red fluorescence), and TRF2 antibody (green fluorescence) ; B. The statistical analysis of the IF experiment results in the figure 1B and figure S2A. The number of co-localized foci of ZBTB40 and TRF2 in a nucleus was counted. The abscissa represented the categories of cells based on the number of foci of ZBTB40 colocalized with TRF2 in a nucleus, while the ordinate indicated the proportion of cells for all cell categories.


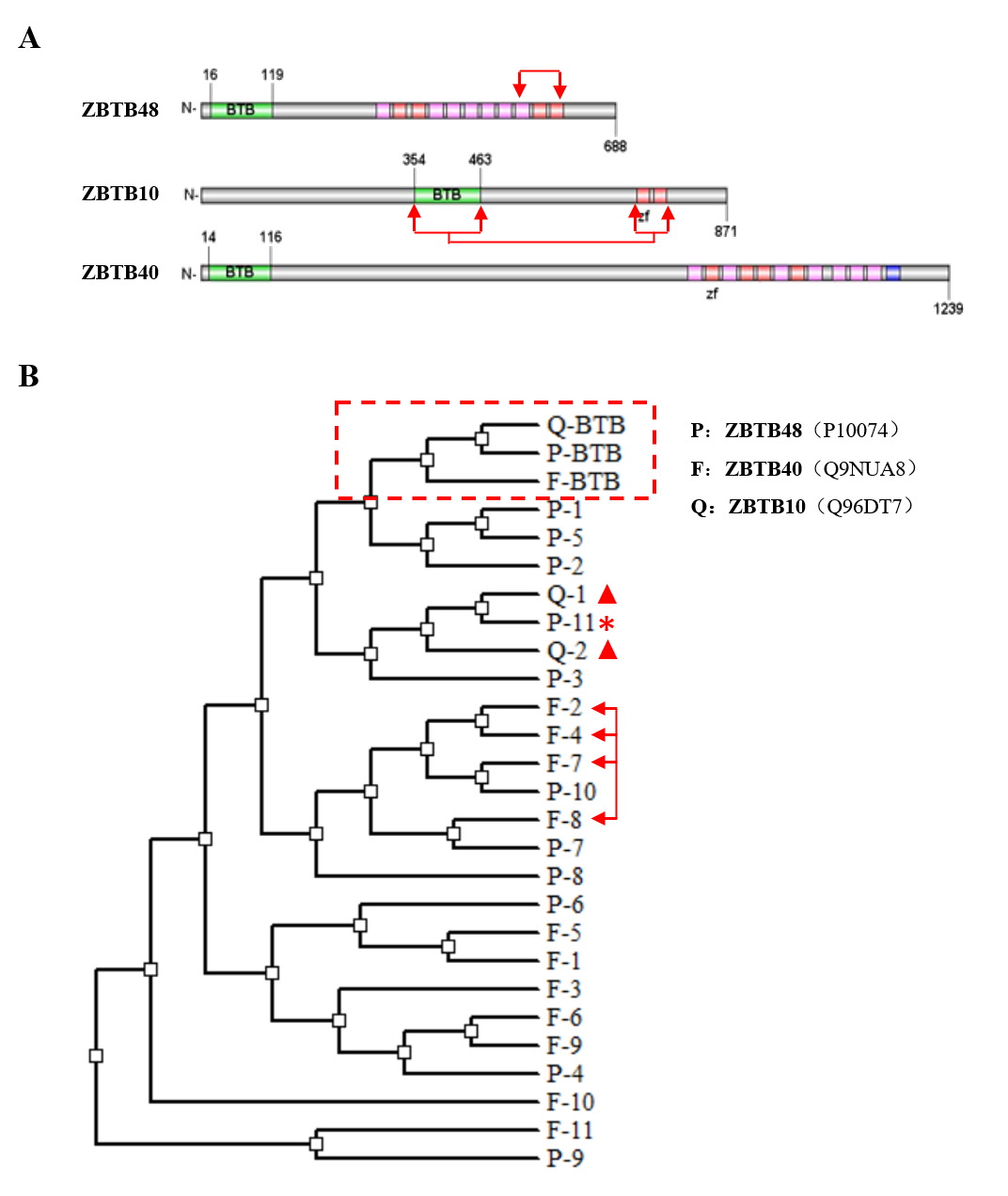


**Figure S3. Comparison of ZBTB40 against ZBTB48 and ZBTB10 in protein domain and sequences.** A. Protein domain architectures of ZBTB40, ZBTB10, and ZBTB48; The red arrows indicated the domains essential for DNA binding; B. Multiple alignments of the sequences of three ZBTB proteins to identify similar domains between ZBTB40 and other proteins. The red triangles and asterisks denoted the zinc fingers of ZBTB10 and ZBTB48, respectively which binds to telomere DNA. The red dash box indicated the BTB domains of three ZBTB proteins with cluster together. The red arrows denoted the four zinc fingers of ZBTB40 that had high sequence similarity to P-10 domain of ZBTB48.


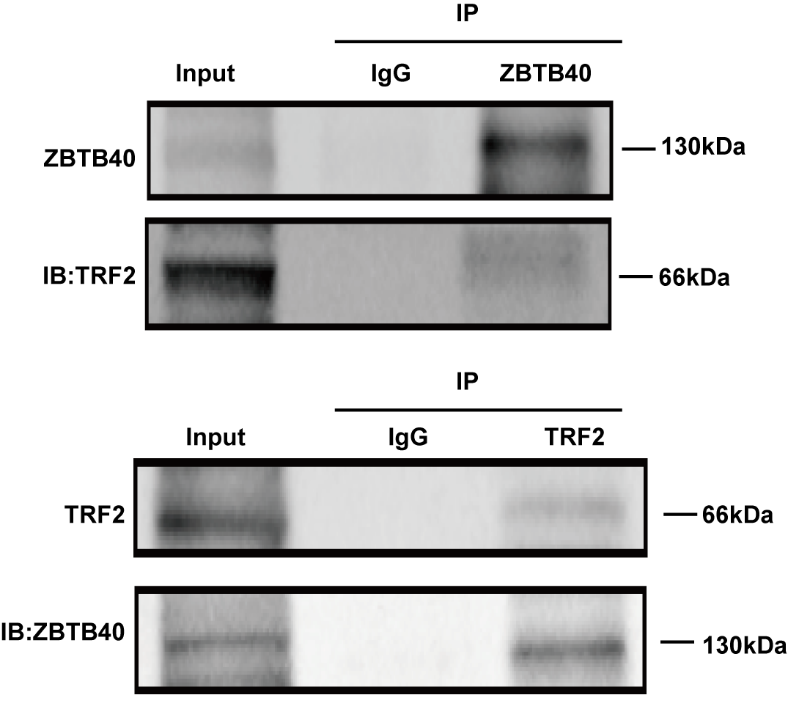


**Figure S4. The Co-IP assays detected the association between ZBTB40 protein and TRF2 (a key sheltering protein) in the of the ALT cells.** The top panel shows the ZBTB40 could pull down TRF2, and the below panel revealed TRF2 was able to bind to ZBTB40.


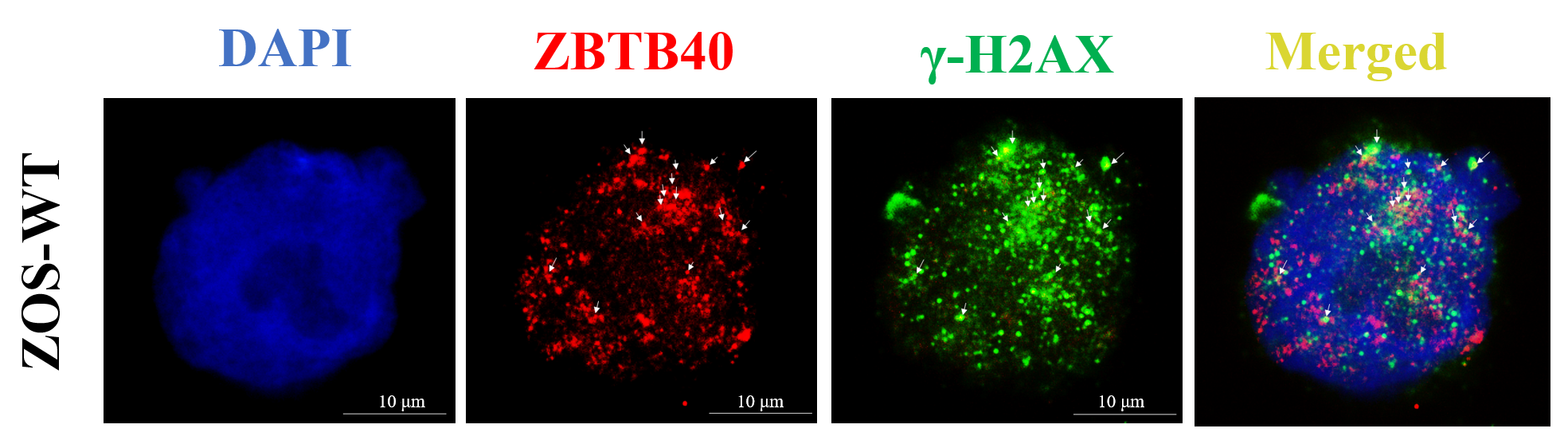


**Figure S5. ZBTB40 is co-localized with γ-H2AX in the ZOS cells.** Representative immunofluorescence results showing co-localization of ZBTB40 and γ-H2AX in the ZOS cells. The nuclei and target proteins were stained with DAPI (blue fluorescence), ZBTB40 (red fluorescence), and γ-H2AX (green fluorescence).


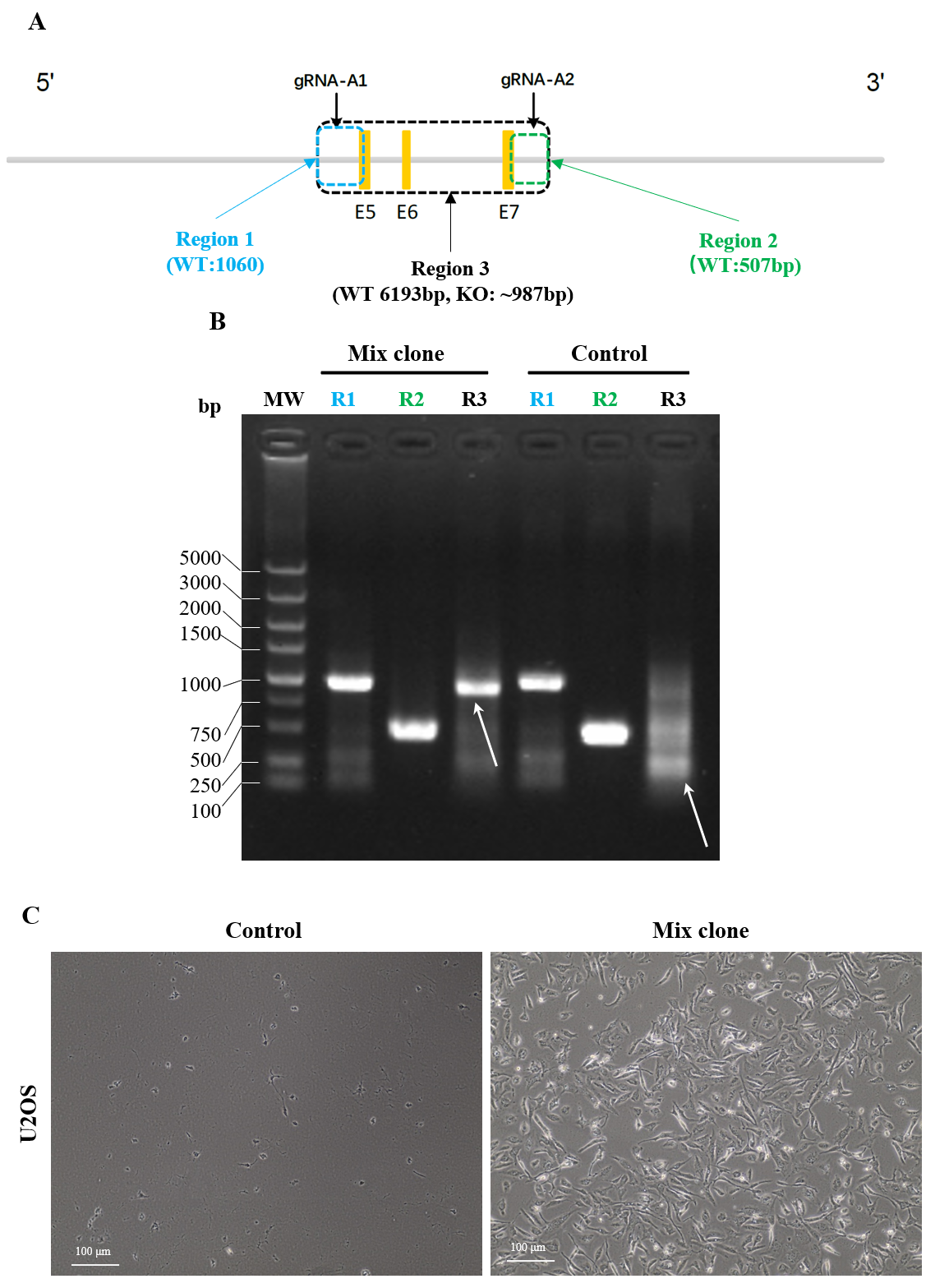


**Figure S6. The generation of the ZBTB40 knockout U2OS cells.** A. The Human ZBTB40 gene (NM_001083621.2) of the U2OS cells was knocked out by electro-transfection mediated CRISPR/CAS9 gene editing. The genomic locations of the two gRNA targeting sites and three PCR validation sites (including the length of PCR products) were shown in the diagram. The sequences of gRNAs and PCR primer could be found at Table S7, S8; B. The ZBTB40 knockout mix clone was validated using PCR; C. The mixed KO clone and control cells were amplified and selected by 2-10 μ g/ μ l puromycin for 7 days.

**
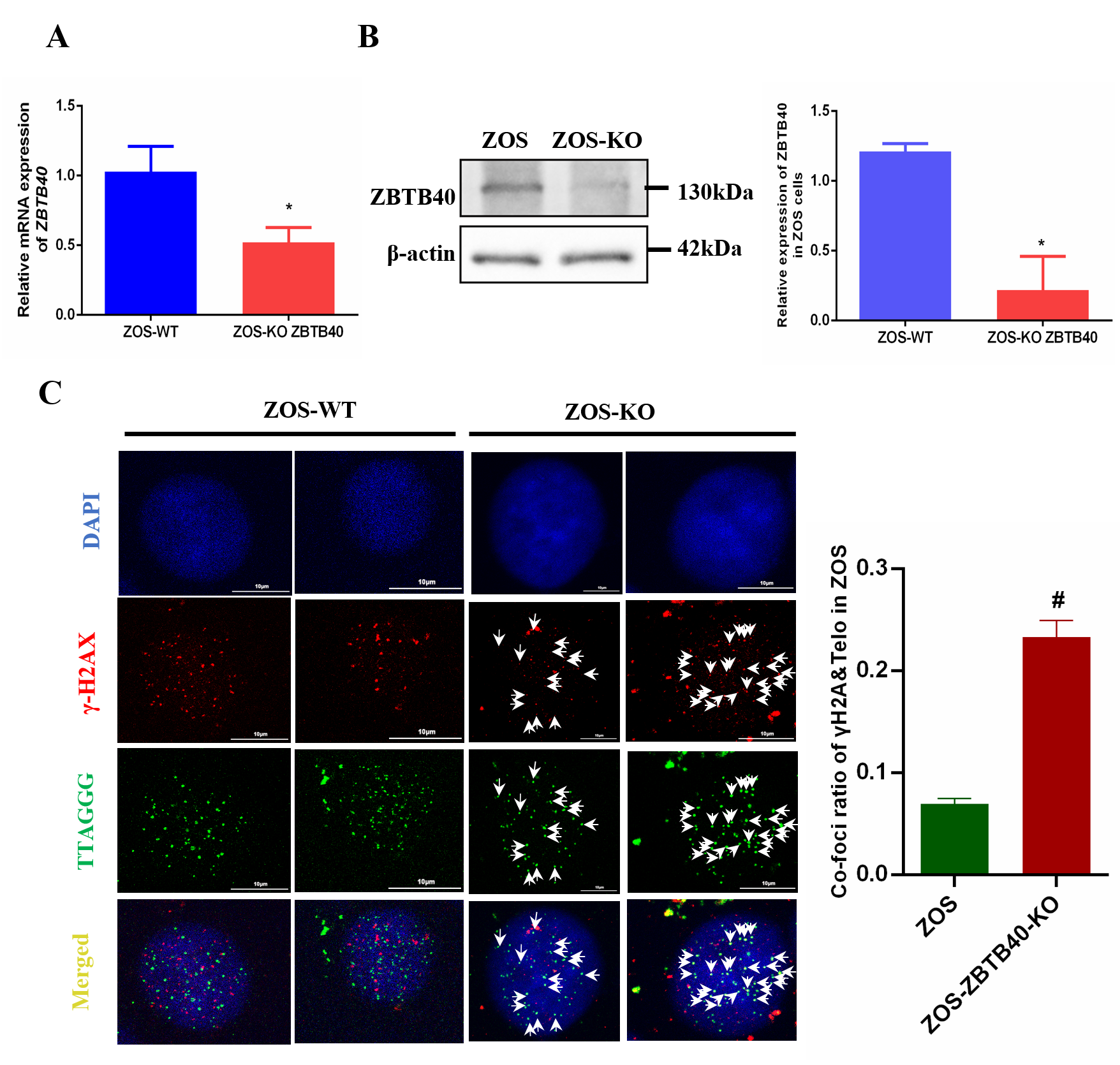
**

**Figure S7. Loss of ZBTB40 leads to telomere dysfunction in the ZOS cells.** A. qRT-PCR assay showed ZBTB40 transcription in ZBTB40 knockdown ZOS cells. B. Western blots revealed ZBTB40 protein level in ZBTB40 knockout ZOS cells; C. FISH showed the TIFs induced by ZBTB40 knockout. Telomeric DNA and γH2A were stained as green fluorescence and red fluorescence, respectively, in the ZOS cells. White arrows indicated the co-localization numbers of γH2A and telomere DNA (TIF) in the ZOS cells. For *p* values of t-tests in all figures: * indicated *p*<0.05, and # denoted *p*<0.01.


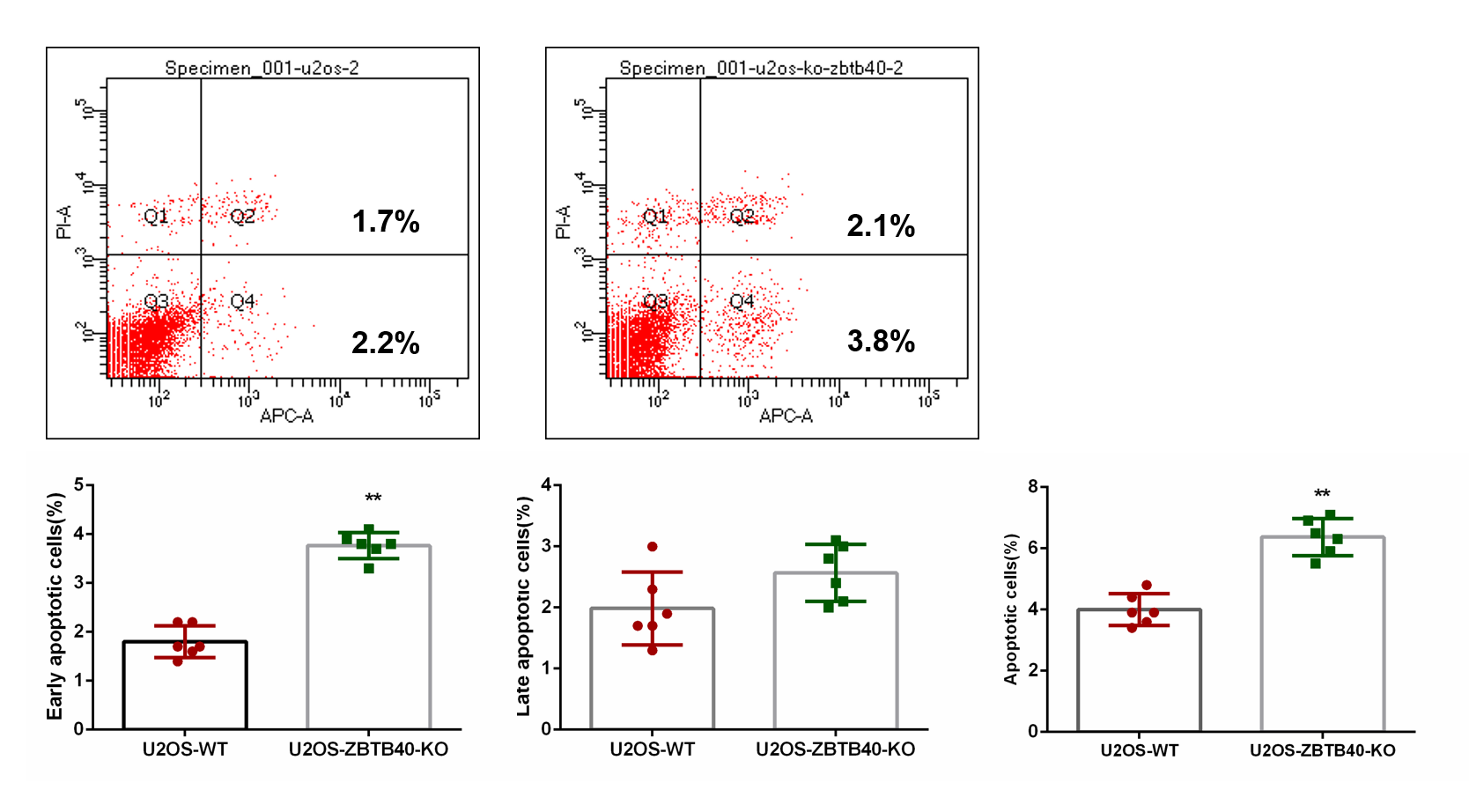


**Figure S8. The cell apoptois ratio of the WT and ZBTB40 defected U2OS cells.** (A) flow cytometry was used to detect the cell apoptosis of WT (left panel) and ZBTB40-KO U2OS cells (right panel). (B) The statistical analysis of the different early apoptosis rate between WT and ZBTB40-KO U2OS cells. (C) The statistical analysis of the different late apoptosis rate between WT and ZBTB40-KO U2OS cells. (D) The statistical analysis of the total apoptosis rate between WT and ZBTB40-KO U2OS cells. For *p* values of t-tests in all figures: **denoted *p*<0.01.


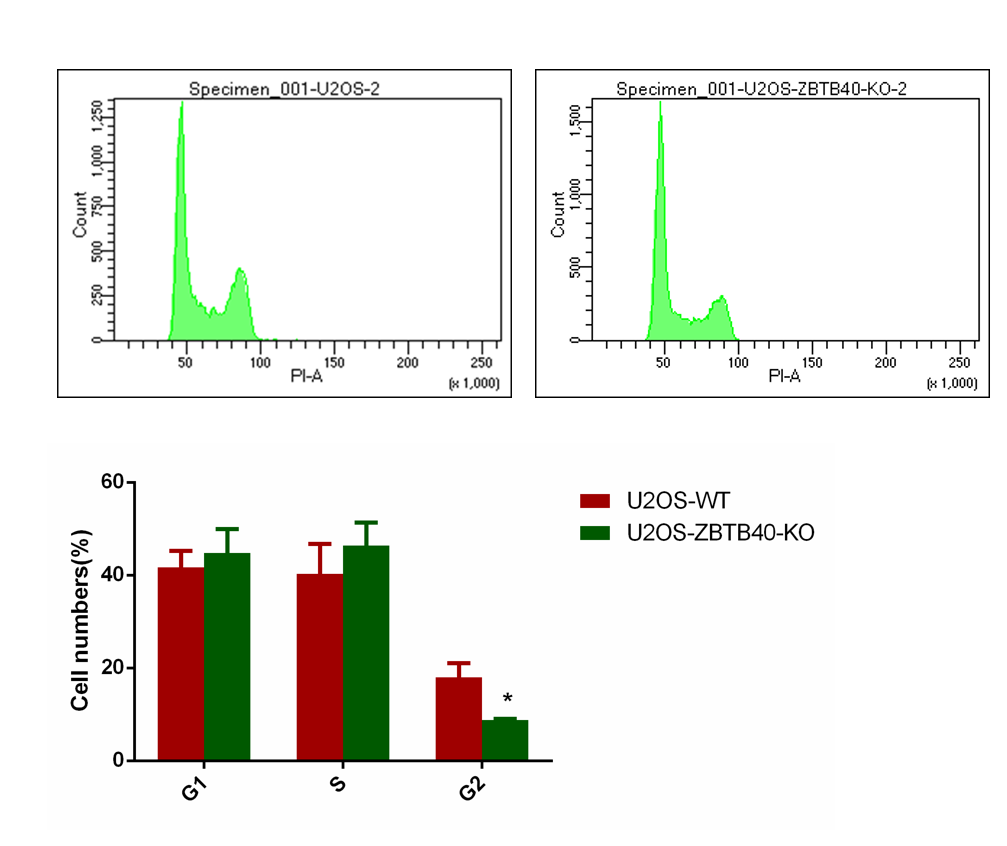


**Figure S9. The cell cycle progress in WT and ZBTB40 defected U2OS cells.** (A) flow cytometry and PI staining were used to detect the cell cycle of WT (left panel) and ZBTB40-KO U2OS cells (right panel). (B) The statistical analysis of the number of G1, S, and G2 cells . For *P* values of t-tests in all figures: *denoted *P* <0.05.


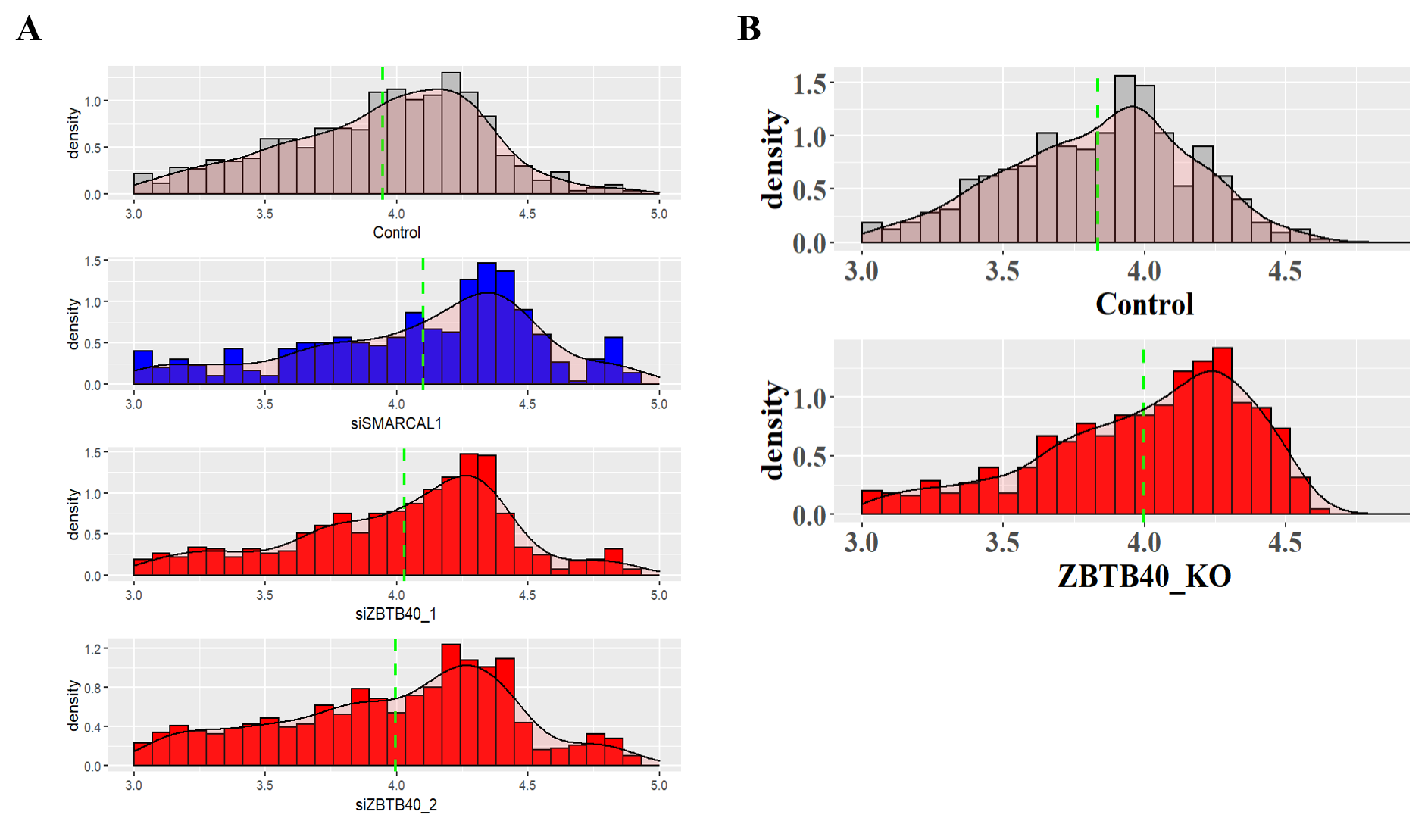


**Figure S10. The distribution of relative telomere length in the ZBTB40 defected U2OS cells.** A. The histograms show the distribution of relative telomere length of M-phase of two U2OS KD cells, siSMARCAL1 and wide type control; B. The histograms illustrate the distribution of relative telomere length of the U2OS KO cells and control U2OS cells in M-phase.


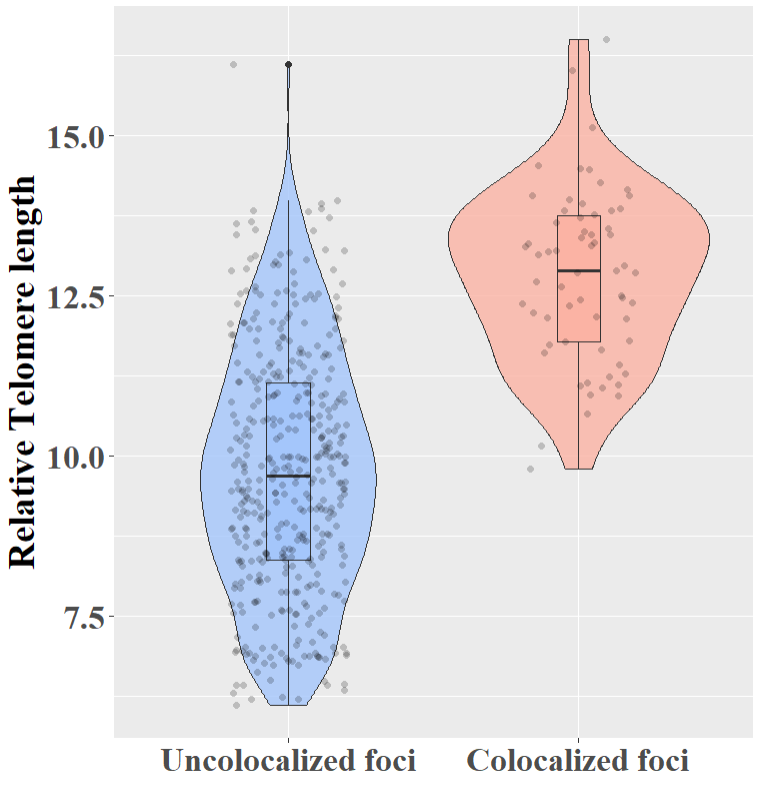


**Figure S11. The distribution of telomere length of telomere foci colocalized with or without ZBTB40 in U2OS cells.** 487 telomeric foci from 9 U2OS cells were divided into two groups (63 colocalized foci and 424 un-colocalized foci). Wilcox test indicates that ZBTB40 colocalized telomeres are significantly longer than their counterparts (P<2.2e-16). The relative telomere length is represented by log2 transformed fluorescence intensity of foci.

**Supplemental Tables 1-9**

**Table S1. Sequences of siRNAs**

| **siRNAs** | **Sequences (5’-3’)** |
| --- | --- |
| **siZBTB40_HSS115018** | **GGA GGA AUU CCU GAC UGG CAC UGA A** |
|  | **UUC AGU GCC AGU CAG GAA UUC CUC C** |
| **siZBTB40_HSS115019** | **GGA AAC GUG GAA GGU GAG UAA UAA A** |
|  | **UUU AUU ACU CAC CUU CCA CGU UUC C** |
| **siZBTB40_HSS115020** | **GCU UCC AUU UCU ACU GCC GCC UAA A** |
|  | **UUU AGG CGG CAG UAG AAA UGG AAG C** |
| **siSMARCAL1HSS121297** | **CCA GGA AGU GGA GCU UUC UCU UGG A** |
|  | **UCC AAG AGA AAG CUC CAC UUC CUG G** |
| **siSMARCAL1HSS121299** | **GGA CAG AAG GAA GAC UCC AGC AGA A** |
|  | **UUC UGC UGG AGU CUU CCU UCU GUC C** |
| **siSMARCAL1HSS121204** | **CAG AAC AUU UCU UAC AUC CAU UCU A** |
|  | **UAG AAU GGA UGU AAG AAA UGU UCU A** |
| **SIC001** | **siRNA Universal Negative Control #1** |

**Table S2. Sequences of telomeric oligonucleotides**

| **Telomere DNA** | **Oligonucleotide sequences 5'-3'** |
| --- | --- |
| **TTAGGG_for** | **TTAGGGTTAGGGTTAGGGTTAGGGTTAGGGTTAGGGTTAGGGTTAGGGTTAGGGTTAGGG** |
| **TTAGGG_rev** | **AACCCTAACCCTAACCCTAACCCTAACCCTAACCCTAACCCTAACCCTAACCCTAACCCT** |

**Table S3. Primers of ZBTB40 mutation constructs**

| **Name** | **Sequences (5'-3')** |
| --- | --- |
| **▲zinc1~11-F** | **AAGAGGCTTCCACCAAAGGTGATG** |
| **▲zinc1~11-R** | **CTTTGGTGGAAGCCTCTTTGGAAGCCTCTTCTT** |
| **▲zinc8~11-F** | **GGAGGGAAGCCCCCAAAGGTGATGAGC** |
| **▲zinc8~11-R** | **CTTTGGGGGCTTCCCTCCGGGCTTCCCTCCAAC** |
| **▲zinc1~7-F** | **AAGAGGCTTCCATTCAGCTGCGGGATC** |
| **▲zinc1~7-R** | **GCTGAATGGAAGCCTCTTTGGAAGCCTCTTCTT** |
| **▲zinc2-F** | **GATGAGAAGCCTTTCATGTGCAAGCAC** |
| **▲zinc2-R** | **CATGAAAGGCTTCTCATCGAAAGGCTTCTCATCGAA** |
| **▲zinc4-F** | **GAAGGGAAAATGTATGTCTGCAGAGAC** |
| **▲zinc4-R** | **GACATACATTTTCCCTTCACATTTTCCCTTCTGAGTG** |
| **▲zinc7-F** | **AAAGAGTACCACTTCAGCTGCGGGATC** |
| **▲zinc7-R** | **GCAGCTGAAGTGGTACTCTTTGGAGTGCAC** |
| **▲BTB-F** | **GAGCAGCAGTTCCACAACTTCTCCAAA** |
| **▲BTB-R** | **GTTGTGGAACTGCTGCTCGAACTGCTGCTCCTT** |
| **▲mid-F** | **TTCTCCAAAATCAAGAAGAAGAGGCTT** |
| **▲mid-R** | **CCTCTTCTTCTTGATTTTGGAGAAGTT** |
| **L4-NotⅠ-FOR** | **AAATATGCGGCCGCTATAAAATGGAGCTCCCCAAC** |
| **L4-AscⅠ-REV** | **TTGGCGCGCCAATCATTTGGCCTCACCACAGAT** |

**Table S4. Primers of Real-Time Quantitative PCR**

| **Primer** | **Sequence (5'-3')** | **Product(bp)** |
| --- | --- | --- |
| **GAPDH F** | **TGAGTACGTCGTGGAGTCCACTG** | 134 |
| **GAPDH R** | **GGTTCACACCCATGACGAAC** |  |
| **ZBTB40 F** | **GGTGTGAACCATGAGAAGTATGA** | 188 |
| **ZBTB40 R** | **GAGTCCTTCCACGATACCAAAG** |  |
| **Gapdh F** | **GCACAGTCAAGGCCGAGAAT** | 151 |
| **Gapdh R** | **GCCTTCTCCATGGTGGTGAA** |  |
| **Zbtb40 F** | **GCAGTACCACAAGCTGACCGA** | 155 |
| **Zbtb40 R** | **TGAAGGTCATGAGGCAATGCT** |  |

**Table S5. Primary antibodies used in this study**

| **Gene** | **Cat. No.#** | **Compaies** | **Assays** | **Host** | **Concentration** |
| --- | --- | --- | --- | --- | --- |
| **ZBTB40** | abs134922-1 | Absin | WB, IHC, IF/ICC, ELISA | Rabbit | WB l:500;IF 1:200; |
| **ZBTB40** | A301-932A | BETHYL | WB, ChIP, IHC, IF, | Rabbit | WB 1:2000;IP 1:500 |
| **γ-H2A** | 05-636-I | Sigma-Aldrich | WB, ICC, ChIP, IF, | Mouse | IF 1:2000;IP 1:2000 |
| **TRF2** | NB100-56506 | Novus | WB, ChIP,IF/ICC,ELISA | Mouse | IF 1:500;IP 1:500 |
| **Flag** | F1840 | SIGMA | WB, IF/ICC, IP, EIA | Mouse | WB l:5000;IF 1:3000; |
| **GST** | 2622 | CST | WB, IP | Rabbit | WB 1:1000;IP 1:100 |
| **GST** | 2624 | CST | WB, IP, IF/ICC | Rabbit | WB 1:1000;IP 1:200;IF 1:3000 |
| **β-actin** | AP0060 | Bioworld | WB | Rabbit | 1:5000 |
| **GAPDH** | TA-08 | Zhongshanjinqiao | WB | Rabbit | 1:2000 |

**Table S6. Secondary antibodies utilized in this study**

| **Name** | **Company** | **cat#** | **Reactivity** |
| --- | --- | --- | --- |
| **Goat Anti-Mouse HRP** | Bio-Rad | 1706516 | Mouse |
| **Goat Anti-Rabbit HRP** | Bio-Rad | 1706515 | Rabbit |
| **Alexa Fluor 488** | CST | 4408S | Mouse |
| **Alexa Fluor 555** | CST | 4413S | Rabbit |
| **Alexa Fluor 594** | CST | 8890S | Mouse |
| **Alexa Fluor 488** | CST | 4412S | Rabbit |

**Table S7. gRNAs used in ZBTB40 knockout U2OS cell generation**

| **Name** | **Sequence (5'-3')** |
| --- | --- |
| **gRNA-A1** | **AAGGTTAGAGCATGGAGTCG TGG** |
| **gRNA-A2** | **ATTAACGTGTGGCTTTCCCA GGG** |

**Table S8. Primers for ZBTB40 knockout U2OS cell validation**

| **Name** | **Sequences (5'-3')** |
| --- | --- |
| **Region_1_F** | **AGGAGTGGTCAGGAAGGCAA** |
| **Region_1_R** | **TATTGGTACAGCCTCAACAGGGA** |
| **Region_2_F** | **TTGCCAAGCACCACAGGTAT** |
| **Region_2_R** | **TGGCTTGAGCTGGATTGTCT** |
| **Region_3_F** | **AGGAGTGGTCAGGAAGGCAA** |
| **Region_3_R** | **GGACAGGGAAGAGTTGGCTT** |

**Table S9. Accession IDs of ChIP-Seq data employed in this study**

| Target Gene | FastQ file ID (ENCODE Database) |
| --- | --- |
| GATAD2A | **ENCFF160GSI;ENCFF076CUB** |
| eGFP-TEAD2 | **ENCFF734FMS;ENCFF335SYB** |
| eGFP-PYGO2 | **ENCFF315YLZ;ENCFF649JEY** |
| SKIL | **ENCFF493LNW;ENCFF310DAJ** |
| FOXA1 | **ENCFF394CJB;ENCFF186GBM** |
| SNRNP70 | **ENCFF466MIW** |
| BCLAF1 | **ENCFF744DBX;ENCFF834VSH** |
| E2F1 | **ENCFF907JIE;ENCFF399CCC** |
| CCAR2 | **ENCFF522MWJ** |
| eGFP-ZNF24 | **ENCFF977AJQ;ENCFF157JAL** |
| RBFOX2 | **ENCFF453JCJ** |
| eGFP-ATF3 | **ENCFF647KQT;ENCFF311SGU** |
| eGFP-MAFG | **ENCFF683PDC;ENCFF307CQO** |
| eGFP-E2F5 | **ENCFF633GWT** |
| DDX20 | **ENCFF881HGO;ENCFF071FDG** |
| eGFP-CEBPG | **ENCFF392MVW;ENCFF567TRK** |
| eGFP-CREB3 | **ENCFF199DAD;ENCFF026WQA** |
| eGFP-RELA | **ENCFF148FMX;ENCFF528GYT** |
| ZC3H8 | **ENCFF172ORX;ENCFF721BBC** |
| eGFP-ETV1 | **ENCFF548SNS;ENCFF898MPN** |
| MCM3 | **ENCFF721WRK;ENCFF501RUA** |
| H3K9ac | **ENCFF000VEG** |
| eGFP-HINFP | **ENCFF551WWR;ENCFF541TDH** |
| POLR2G | **ENCFF355VVD** |
| H3K4me3 | **ENCFF000VDX** |
| EGR1 | **ENCFF025AGM;ENCFF751XCN** |
| ETV6 | **ENCFF696VXH** |
| ESRRA | **ENCFF002DUB;ENCFF002DOY** |
| HES1 | **ENCFF583IRH;ENCFF600EDO** |
| NCOA1 | **ENCFF165NJF;ENCFF226TLO** |
| SMAD5 | **ENCFF001RUM** |
| NFE2 | **ENCFF001RUN** |
| MITF | **ENCFF001RUI** |
| TRIM24 | **ENCFF107WKN;ENCFF950QCU** |
| ZSCAN29 | **ENCFF684YTI;ENCFF412VSI** |
| ESRRA | **ENCFF002DOX;ENCFF002EFV** |
| eGFP-IRF1 | **ENCFF031RWN;ENCFF031WIT** |
| eGFP-ID3 | **ENCFF548RTA;ENCFF916QKA** |
| KDM1A | **ENCFF147CXN;ENCFF408SWF** |
| H3K4me1 | **ENCFF000BXX** |
| eGFP-ILK | **ENCFF908PTR;ENCFF007IDQ** |
| NRF1 | **ENCFF002EFZ;ENCFF002DPF** |
| SRSF1 | **ENCFF025BOT** |
| RNF2 | **ENCFF470MPB;ENCFF742JMT** |
| eGFP-NR2C1 | **ENCFF960ZID;ENCFF096VXL** |
| eGFP-FOXJ2 | **ENCFF432PNM** |
| MGA | **ENCFF434ISC;ENCFF105BAY** |
| H3K27ac | **ENCFF000BXH** |
| HDAC2 | **ENCFF990UUV;ENCFF741IHV** |
| eGFP-ZFX | **ENCFF215QBP** |
| H3K4me3 | **ENCFF010SAE** |
| TEAD4 | **ENCFF000QIS** |
| eGFP-HDAC8 | **ENCFF393ANW;ENCFF351UCM** |
| STAT5A | **ENCFF000QID** |
| CREB1 | **ENCFF000PXY** |
| eGFP-GATA2 | **ENCFF000ZUK** |
| RFX1 | **ENCFF493EZE;ENCFF207YQO** |
| eGFP-JUND | **ENCFF000ZUU** |
| eGFP-JUNB | **ENCFF000ZUR** |
| ZZZ3 | **ENCFF960PEL;ENCFF491EOP** |
| GATAD2B | **ENCFF552ETP;ENCFF523ULM** |
| eGFP-CREB3 | **ENCFF199DAD;ENCFF026WQA** |
| E2F1 | **ENCFF907JIE;ENCFF399CCC** |
| eGFP-NR2C1 | **ENCFF960ZID;ENCFF096VXL** |
| CTBP1 | **ENCFF829YQD;ENCFF077DIB** |
| L3MBTL2 | **ENCFF945QMW;ENCFF139CLF** |
| SIN3B | **ENCFF526SSV;ENCFF953OIR** |
| JUNB | **ENCFF100SGJ** |
| SAP30 | **ENCFF873QQB** |
| RB1 | **ENCFF615SOF;ENCFF656UEH** |
| ZBTB33 | **ENCFF332XKO;ENCFF189UUN** |
| RNF2 | **ENCFF470MPB;ENCFF742JMT** |
| EGR1 | **ENCFF025AGM;ENCFF751XCN** |
| EP400 | **ENCFF076RPA;ENCFF636OJT** |
| CCNT2 | **ENCFF000YHX** |
| ZZZ3 | **ENCFF960PEL;ENCFF491EOP** |
| eGFP-TAF7 | **ENCFF926UQW;ENCFF098GTH** |
| CSDE1 | **ENCFF940EGQ;ENCFF255NDV** |
| LARP7 | **ENCFF324CSR;ENCFF351HKA** |
| eGFP-NFE2 | **ENCFF118PSY;ENCFF034LKJ** |
| eGFP-MAFG | **ENCFF683PDC;ENCFF307CQO** |
| eGFP-ZBTB11 | **ENCFF112KAJ** |
| eGFP-DDX20 | **ENCFF706WJQ;ENCFF077SBC** |
| RAD51 | **ENCFF354UBV;ENCFF600KXI** |
| ZNF24 | **ENCFF931UCZ;ENCFF965QBT** |
| NKRF | **ENCFF599KBS;ENCFF259JYL** |
| HDAC1 | **ENCFF677ULQ;ENCFF410PAM** |
| ATF7 | **ENCFF002DOR;ENCFF002EIJ** |
| STAT5A | **ENCFF000QID** |
| MEIS2 | **ENCFF002EIW;ENCFF002EIX** |
| CREB3L1 | **ENCFF002EIT;ENCFF002EIQ** |
| CREB3L1 | **ENCFF002EIT;ENCFF002EIQ** |
| eGFP-KLF1 | **ENCFF994DFN;ENCFF438UFH** |
| LEF1 | **ENCFF211HML;ENCFF238ZLM** |
| NFATC3 | **ENCFF483YZA;ENCFF570GLE** |
| MEIS2 | **ENCFF002EIW;ENCFF002EIX** |
| BCOR | **ENCFF423KKF;ENCFF047XMV** |
| eGFP-MAFG | **ENCFF683PDC;ENCFF307CQO** |
| eGFP-ETS2 | **ENCFF466RJA;ENCFF548XLM** |
| NFATC3 | **ENCFF483YZA;ENCFF570GLE** |
| eGFP-CEBPB | **ENCFF470OUZ;ENCFF555QJQ** |
| ELK1 | **ENCFF000YMO** |
| MCM3 | **ENCFF721WRK;ENCFF501RUA** |
| NFXL1 | **ENCFF358LFL;ENCFF575GFV** |
| eGFP-GTF2A2 | **ENCFF677HTA;ENCFF339NHI** |
| CHAMP1 | **ENCFF088KVK;ENCFF576YEJ** |
| SMARCE1 | **ENCFF034ESF;ENCFF668WFT** |
| TAF7 | **ENCFF000QIM** |
| eGFP-JUND | **ENCFF000ZUU** |
| eGFP-KLF13 | **ENCFF358PYP;ENCFF684JBQ** |
| eGFP-CEBPB | **ENCFF156EZY;ENCFF052XOQ** |
| THAP1 | **ENCFF000QIY** |
| eGFP-JUNB | **ENCFF000ZUR** |
| SRSF7 | **ENCFF161FHO** |
| TEAD4 | **ENCFF000QIW** |
| eGFP-GATA2 | **ENCFF000ZUK** |
| SMAD1 | **ENCFF002EBF** |
| HMBOX1 | **ENCFF705WBN** |
| eGFP-CEBPG | **ENCFF496CZA;ENCFF028NOQ** |
| Control | **ENCFF417ORB;ENCFF839JCV** |
| CSDE1 | **ENCFF940EGQ;ENCFF255NDV** |
| SMAD2 | **ENCFF002EBV** |
| TAL1 | **ENCFF998YDA;ENCFF768SQO** |
| PHB2 | **ENCFF499ADG;ENCFF005PFF** |
| eGFP-CUX1 | **ENCFF320OTV** |
| ZBTB11 | **ENCFF052ZGR** |
| eGFP-IRF9 | **ENCFF137GVE;ENCFF991EAW** |
| eGFP-IRF9 | **ENCFF034WFD;ENCFF183THJ** |
| FOXK2 | **ENCFF260CTF;ENCFF959CFI** |
| TAL1 | **ENCFF998YDA;ENCFF768SQO** |
| ATF3 | **ENCFF367HUF;ENCFF169CRK** |
| DPF2 | **ENCFF952UEN;ENCFF245SAZ** |
| GATA2 | **ENCFF000QBD** |
| KLF16 | **ENCFF855OMG** |
| eGFP-NR2C1 | **ENCFF960ZID;ENCFF096VXL** |
| CBX5 | **ENCFF002BEZ** |
| MAZ | **ENCFF000YTS** |
| POLR3A | **ENCFF000YYW** |
| eGFP-DDX20 | **ENCFF706WJQ;ENCFF077SBC** |
| eGFP-ETV1 | **ENCFF548SNS;ENCFF898MPN** |
| IRF1 | **ENCFF000YRS** |
| eGFP-HINFP | **ENCFF423ZOG;ENCFF224KMR** |
| ZNF592 | **ENCFF283GNM;ENCFF451RCX** |
| ZNF407 | **ENCFF864KXI;ENCFF678SZB** |
| eGFP-MEF2D | **ENCFF703DNA;ENCFF958CZA** |
| POLR2A | **ENCFF839LPL;ENCFF698ICA** |
| eGFP-PYGO2 | **ENCFF315YLZ;ENCFF649JEY** |
| ZC3H11A | **ENCFF000ZDS** |
| eGFP-NR4A1 | **ENCFF671VDI;ENCFF301GRJ** |
| eGFP-ZNF584 | **ENCFF947GWY** |
| eGFP-PBX2 | **ENCFF932THV;ENCFF410UIX** |
| eGFP-HDAC8 | **ENCFF393ANW;ENCFF351UCM** |
| ZNF184 | **ENCFF880UNP;ENCFF264CHQ** |
| USF2 | **ENCFF000ZDJ** |
| KDM4B | **ENCFF571BGG;ENCFF686DFA** |
| MYNN | **ENCFF796BOA** |
| eGFP-ZNF512 | **ENCFF123LEB;ENCFF229NRD** |
| POU5F1 | **ENCFF002EEQ** |
| SREBF1 | **ENCFF002EEP** |
| ZHX1 | **ENCFF002EET** |
| eGFP-POLR2H | **ENCFF613UAT;ENCFF618IBA** |
| IRF2 | **ENCFF002EEC** |
| ARID2 | **ENCFF991XUU;ENCFF385PER** |
| NCOA1 | **ENCFF165NJF;ENCFF226TLO** |
| TARDBP | **ENCFF996BVX;ENCFF654EXQ** |
| CEBPZ | **ENCFF002EEI** |
| NEUROD1 | **ENCFF002EEO** |
| eGFP-TAF7 | **ENCFF926UQW;ENCFF098GTH** |
| eGFP-DIDO1 | **ENCFF382BWE;ENCFF943GMA** |
| HNRNPL | **ENCFF698TRM** |
| eGFP-HINFP | **ENCFF423ZOG;ENCFF224KMR** |
| NR2F6 | **ENCFF976LPV** |
| eGFP-ELF1 | **ENCFF073VHT** |
| SRSF3 | **ENCFF137DST** |
| XRCC5 | **ENCFF925UZZ** |
| eGFP-NFE2L1 | **ENCFF576NTQ;ENCFF686CXJ** |
| ARNT | **ENCFF546YEQ;ENCFF446XHP** |
| FOXK2 | **ENCFF260CTF;ENCFF959CFI** |
| SPI1 | **ENCFF000QED** |
| eGFP-HDAC8 | **ENCFF393ANW;ENCFF351UCM** |
| ZNF316 | **ENCFF308CZX;ENCFF786NXV** |
| SFPQ | **ENCFF576NBS** |
| ZKSCAN1 | **ENCFF002EWL** |
| ZNF316 | **ENCFF308CZX;ENCFF786NXV** |
| RAD21 | **ENCFF000QEJ** |
| TCF7L2 | **ENCFF002EWU** |
| YY1 | **ENCFF000QKF** |
| eGFP-ZNF83 | **ENCFF471BMT** |
| TOE1 | **ENCFF831SWK;ENCFF738RCP** |
| eGFP-PTRF | **ENCFF474LQU;ENCFF532XZW** |
| USF1 | **ENCFF000QJP** |
| CTCFL | **ENCFF000PYR** |
| EGR1 | **ENCFF871VWH;ENCFF045QIG** |
| BRCA1 | **ENCFF276RGL;ENCFF231TGV** |
| MAFK | **ENCFF000YUE** |
| NFXL1 | **ENCFF358LFL;ENCFF575GFV** |
| ZBTB7A | **ENCFF000QLG** |
| eGFP-DIDO1 | **ENCFF597HDK;ENCFF992IHE** |
| eGFP-POLR2H | **ENCFF613UAT;ENCFF618IBA** |
| XRCC5 | **ENCFF925UZZ** |
| MBD2 | **ENCFF837CJW** |
| BHLHE40 | **ENCFF000YGJ** |
| FOXM1 | **ENCFF888ZYM;ENCFF749GVI** |
| NBN | **ENCFF321LCC;ENCFF581MDG** |
| NFRKB | **ENCFF705WPL;ENCFF139VNK** |
| eGFP-USF2 | **ENCFF141CZI** |
| MCM2 | **ENCFF516QHN;ENCFF943CAF** |
| CHAMP1 | **ENCFF088KVK;ENCFF576YEJ** |
| eGFP-GABPA | **ENCFF346WZR** |
| NR2F1 | **ENCFF394OKZ;ENCFF579DKO** |
| ZNF316 | **ENCFF308CZX;ENCFF786NXV** |
| H3K79me2 | **ENCFF000BYO** |
| ZEB2 | **ENCFF776VEC;ENCFF824ADC** |
| H3K4me2 | **ENCFF000BYF** |
| SFPQ | **ENCFF576NBS** |
| eGFP-ZNF507 | **ENCFF306MYT;ENCFF574SDW** |
| H3K9me1 | **ENCFF000BYY** |
| ZEB2 | **ENCFF776VEC;ENCFF824ADC** |
| PRPF4 | **ENCFF984DIG** |
| eGFP-NFE2L1 | **ENCFF576NTQ;ENCFF686CXJ** |
| CBX2 | **ENCFF000BVN** |
| KDM1A | **ENCFF147CXN;ENCFF408SWF** |
| CTCF | **ENCFF000YLW** |
| CREBBP | **ENCFF000BVH** |
| eGFP-GTF2E2 | **ENCFF428TYZ** |
| ZBTB40 | **ENCFF520UHF;ENCFF687MII** |
| RCOR1 | **ENCFF000YLH** |
| eGFP-NFE2L1 | **ENCFF576NTQ;ENCFF686CXJ** |
| eGFP-KLF1 | **ENCFF994DFN;ENCFF438UFH** |
| eGFP-MEF2D | **ENCFF703DNA;ENCFF958CZA** |
| ZBTB33 | **ENCFF332XKO;ENCFF189UUN** |
| KAT8 | **ENCFF505YGU;ENCFF093OLE** |
| RB1 | **ENCFF615SOF;ENCFF656UEH** |
| HNRNPLL | **ENCFF218JDY** |
| FOXA1 | **ENCFF394CJB;ENCFF186GBM** |
| COPS2 | **ENCFF610FSQ;ENCFF064WKY** |
| MTA3 | **ENCFF894NUI;ENCFF809IJU** |
| MYBL2 | **ENCFF329ODS;ENCFF765DMW** |
| EGR1 | **ENCFF871VWH;ENCFF045QIG** |
| PRPF4 | **ENCFF308HCL** |
| ZBTB5 | **ENCFF865YPR;ENCFF543AIK** |
| CBFA2T3 | **ENCFF238IYZ;ENCFF203LDU** |
| eGFP-DDX20 | **ENCFF706WJQ;ENCFF077SBC** |
| eGFP-NR2C1 | **ENCFF960ZID;ENCFF096VXL** |
| eGFP-ZNF740 | **ENCFF554XIX;ENCFF111REI** |
| eGFP-ZNF507 | **ENCFF306MYT;ENCFF574SDW** |
| eGFP-ID3 | **ENCFF548RTA;ENCFF916QKA** |
| eGFP-BACH1 | **ENCFF979UQU** |
| eGFP-ZNF395 | **ENCFF479NRX** |
| eGFP-ILK | **ENCFF908PTR;ENCFF007IDQ** |
| EP400 | **ENCFF076RPA;ENCFF636OJT** |
| CBX3 | **ENCFF000PXA** |
| GABPA | **ENCFF000QAV** |
| RBM17 | **ENCFF614UID** |
| eGFP-ATF1 | **ENCFF018HQR;ENCFF016FNK** |
| CEBPB | **ENCFF000PXH** |
| CEBPD | **ENCFF000PXW** |
| PHF21A | **ENCFF282GIL;ENCFF356VYM** |
| eGFP-PTTG1 | **ENCFF349DXW;ENCFF660MJG** |
| SMC3 | **ENCFF000YZY** |
| ETS1 | **ENCFF000QAG** |
| TARDBP | **ENCFF996BVX;ENCFF654EXQ** |
| eGFP-DDX20 | **ENCFF706WJQ;ENCFF077SBC** |
| FOSL1 | **ENCFF000QAN** |
| THRA | **ENCFF097JYZ;ENCFF499VMV** |
| SAFB | **ENCFF135WKL** |
| NBN | **ENCFF321LCC;ENCFF581MDG** |
| eGFP-ZNF24 | **ENCFF718HUU;ENCFF615CRG** |
| ELF1 | **ENCFF932JXG;ENCFF070OBA** |
| HNRNPH1 | **ENCFF753OYD** |
| ZNF407 | **ENCFF864KXI;ENCFF678SZB** |
| MGA | **ENCFF434ISC;ENCFF105BAY** |
| TBP | **ENCFF000ZCA** |
| MCM2 | **ENCFF516QHN;ENCFF943CAF** |
| eGFP-MAFG | **ENCFF683PDC;ENCFF307CQO** |
| eGFP-ZNF24 | **ENCFF718HUU;ENCFF615CRG** |
| LEF1 | **ENCFF211HML;ENCFF238ZLM** |
| eGFP-NFE2L1 | **ENCFF576NTQ;ENCFF686CXJ** |
| DNMT1 | **ENCFF436CBC;ENCFF306GVI** |
| MCM5 | **ENCFF447WZH;ENCFF238TRM** |
| MTA2 | **ENCFF693GDA;ENCFF259AYK** |
| H3K36me3 | **ENCFF000BXR** |
| ZBTB40 | **ENCFF520UHF;ENCFF687MII** |
| DEAF1 | **ENCFF095DPD** |
| TCF12 | **ENCFF007HDS;ENCFF873EXA** |
| ZBTB5 | **ENCFF865YPR;ENCFF543AIK** |
| ESRRA | **ENCFF002DUB;ENCFF002DOY** |
| HES1 | **ENCFF583IRH;ENCFF600EDO** |
| eGFP-ETS2 | **ENCFF466RJA;ENCFF548XLM** |
| H3K4me3 | **ENCFF010SAE** |
| eGFP-ADNP | **ENCFF652IDC** |
| TOE1 | **ENCFF831SWK;ENCFF738RCP** |
| eGFP-CREB3 | **ENCFF199DAD;ENCFF026WQA** |
| eGFP-KLF13 | **ENCFF519FTA;ENCFF607CQM** |
| SIRT6 | **ENCFF000CCK** |
| MNT | **ENCFF678PWC;ENCFF627YOB** |
| eGFP-ZNF354B | **ENCFF132GDV;ENCFF407ICD** |
| SETDB1 | **ENCFF000CCG** |
| FOXM1 | **ENCFF888ZYM;ENCFF749GVI** |
| PHB2 | **ENCFF499ADG;ENCFF005PFF** |
| SUZ12 | **ENCFF000CCP** |
| CUX1 | **ENCFF000YHM** |
| H4K20me1 | **ENCFF000BZN** |
| SP2 | **ENCFF000QHG** |
| eGFP-KLF13 | **ENCFF519FTA;ENCFF607CQM** |
| eGFP-ZNF644 | **ENCFF922XIL** |
| TAF1 | **ENCFF000QHW** |
| ATF3 | **ENCFF367HUF;ENCFF169CRK** |
| E2F7 | **ENCFF124KEV;ENCFF584BPF** |
| KDM4B | **ENCFF571BGG;ENCFF686DFA** |
| PKNOX1 | **ENCFF755HHM;ENCFF292YNE** |
| TBL1XR1 | **ENCFF000ZBJ** |
| ARID2 | **ENCFF991XUU;ENCFF385PER** |
| eGFP-ILK | **ENCFF908PTR;ENCFF007IDQ** |
| eGFP-PTTG1 | **ENCFF349DXW;ENCFF660MJG** |
| SIN3B | **ENCFF526SSV;ENCFF953OIR** |
| eGFP-ETV1 | **ENCFF548SNS;ENCFF898MPN** |
| BRD4 | **ENCFF049ACQ** |
| H3K27me3 | **ENCFF000VDP** |
| H3K4me3 | **ENCFF894KBP** |
| CHD4 | **ENCFF000BWE** |
| ZNF316 | **ENCFF121HAM;ENCFF890FRG** |
| eGFP-HDAC8 | **ENCFF393ANW;ENCFF351UCM** |
| eGFP-PTTG1 | **ENCFF349DXW;ENCFF660MJG** |
| KHSRP | **ENCFF323SPJ;ENCFF437GIK** |
| SRSF3 | **ENCFF614XZH** |
| PHF8 | **ENCFF000CBB** |
| eGFP-ILK | **ENCFF908PTR;ENCFF007IDQ** |
| NCOA6 | **ENCFF956KKU;ENCFF957QWK** |
| NR2F2 | **ENCFF000QCS** |
| CBX8 | **ENCFF942JXG;ENCFF388TMG** |
| NCOR1 | **ENCFF224PWB;ENCFF337XLT** |
| SMARCC2 | **ENCFF809LBJ;ENCFF147AFV** |
| KMT2B | **ENCFF792XQD** |
| NR2C1 | **ENCFF146EKI;ENCFF102DIC** |
| HLTF | **ENCFF787PGA;ENCFF017GMQ** |
| eGFP-ZBTB40 | **ENCFF709ELM** |
| JUN | **ENCFF000YJL** |
| ZBTB2 | **ENCFF796XXQ;ENCFF920GKJ** |
| eGFP-ETV1 | **ENCFF548SNS;ENCFF898MPN** |
